# 3D MR Fingerprinting for Quantitative Bladder Wall T_1_, T_2_, and M_0_ Mapping in Healthy Subjects at 1.5 T and 3 T

**DOI:** 10.1002/nbm.70366

**Published:** 2026-07-28

**Authors:** Shane A. Wells, Giulia M. Ippolito, Melis Ozkan, Wilson Sui, Anne P. Cameron, Jesse I. Hamilton

**Affiliations:** ^1^ Department of Radiology University of Michigan Ann Arbor Michigan USA; ^2^ Department of Urology University of Michigan Ann Arbor Michigan USA; ^3^ Department of Biomedical Engineering University of Michigan Ann Arbor Michigan USA

**Keywords:** bladder, deep image prior, deep learning, MR fingerprinting, T_1_ mapping, T_2_ mapping

## Abstract

Current diagnostic tests for lower urinary tract symptoms (LUTS) do not assess tissue‐level alterations in the bladder wall. This study assesses the feasibility of 3D magnetic resonance fingerprinting (MRF) for simultaneous T_1_, T_2_, and proton density (M_0_) mapping of the bladder wall in healthy subjects at 1.5 T and 3 T. A 3D MRF acquisition was combined with a deep image prior reconstruction to achieve whole‐bladder T_1_, T_2_, and M_0_ mapping in 11.8 min at an acquired voxel size of 1 × 1 × 3 mm^3^ (interpolated to 0.5 × 0.5 × 1.5 mm^3^). Twenty‐nine healthy subjects (14 female, 18–78 years) were enrolled, with 16 scanned at both 1.5 T and 3 T. Conventional maps were acquired using MOLLI and T_2_‐prepared GRE sequences. Maps were analyzed by measuring mean global and regional T_1_ and T_2_ values, with spatial heterogeneity assessed using the coefficient of variation (CV). Bladder wall thickness (BWT) was measured from synthetic weighted images derived from MRF maps, compared to reference values from conventional T_2_‐weighted MRI. Mean global MRF bladder wall measurements were 984 ± 53 ms (T_1_), 51.4 ± 5.6 ms (T_2_), and 4.5 ± 1.6 mm (BWT) at 1.5 T and 1468 ± 118 ms (T_1_), 43.5 ± 4.0 ms (T_2_), and 4.3 ± 1.6 mm (BWT) at 3 T. MRF T_1_ values were significantly lower than MOLLI at 1.5 T (bias −151 ms) but not significantly different at 3 T. MRF T_2_ values were significantly lower (by 7–8 ms) than T_2_‐prepared GRE at both field strengths. MRF exhibited lower CV than MOLLI T_1_ mapping at 1.5 T. BWT from synthetic images exhibited excellent agreement (bias ≤0.1 mm) with conventional T_2_‐weighted images. Regionally, T_1_ and T_2_ tended to be higher in the dome and posterior wall. In an exploratory analysis, a positive association between age and T_2_ was observed. In conclusion, 3D MRF of the bladder wall is feasible in healthy subjects, providing a baseline for translational studies to characterize pathological remodeling in LUTS.

## Graphical abstract

Current diagnostic tests for lower urinary tract symptoms lack the ability to assess tissue‐level alterations in the bladder wall. This study introduces an approach for simultaneous high‐resolution 3D T_1_, T_2_, and proton density mapping of the bladder wall using magnetic resonance fingerprinting combined with a self‐supervised deep learning reconstruction. Technical feasibility is demonstrated in healthy subjects scanned in back‐to‐back MRI exams at 1.5 T and 3 T to characterize bladder wall tissue property measurements at both field strengths.

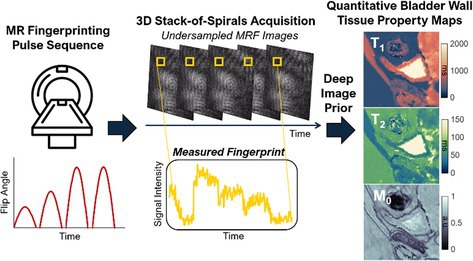

**Abbreviations**
ANOVA: analysis of variance
bSSFP: balanced steady‐state free precession
BWT: bladder wall thickness
CV: coefficient of variation
DIP: deep image prior
GRE: gradient echo
FISP: fast imaging with steady‐state precession
FOV: field of view
FSE: fast spin echo
IPSS: International Prostate Symptom Score
LURN: Lower Urinary Tract Dysfunction Research Network
LUTD: lower urinary tract dysfunction
LUTS: lower urinary tract symptoms
MRF: magnetic resonance fingerprinting
MOLLI: modified Look–Locker inversion recovery
PSIR: phase‐sensitive inversion recovery
PVR: post‐void residual
RF: radiofrequency
ROI: region of interest
ROVir: region‐optimized virtual coils
SD: standard deviation
SNR: signal‐to‐noise ratio
SVD: singular value decomposition
TE: echo time
TI: inversion time
TR: repetition time
UDS: urodynamic studies

## 1 Introduction

Lower urinary tract symptoms (LUTS) are among the most common reasons for urological consultation. More than 60% of adults over age 40 experience LUTS, with prevalence and severity increasing with age [1], and these symptoms impose substantial physical, psychological, and economic burdens [2]. Clinical evaluation currently relies on a combination of patient‐reported measures, functional testing, structural imaging, and cystoscopy, but each has limitations. Validated questionnaires are commonly used to quantify symptom burden but are inherently subjective [3, 4]. Non‐invasive functional testing, typically combining uroflowmetry with ultrasound measurements of post‐void residual (PVR), provides no information about storage symptoms and cannot identify underlying causes of impaired voiding [5]. Urodynamic studies (UDS) remain the reference standard for evaluating detrusor function but are invasive, not recommended for routine evaluation, and influenced by patient and procedural factors that limit reproducibility [6, 7]. Anatomic imaging (ultrasound or CT) and cystoscopy permit structural evaluation of the lower urinary tract but are insensitive to tissue remodeling that may underlie LUTS.

Bladder wall remodeling is increasingly recognized as a major contributor to LUTS. Changes in urothelial permeability, mechanical stress, abnormal neural input, or ischemia may promote bladder wall inflammation, detrusor hypertrophy, and interstitial fibrosis [8, 9]. These alterations reduce bladder compliance during filling and impair contractility during voiding [10]. Several studies have linked collagen deposition and fibrosis with impaired bladder function [10, 11, 12], and elevated bladder wall inflammation and fibrosis have been associated with reduced efficacy of botulinum toxin treatment for neurogenic detrusor overactivity [13, 14]. Accordingly, non‐invasive characterization of bladder wall remodeling could be valuable for early disease detection and treatment monitoring. However, routine bladder biopsy is not recommended for functional urologic conditions, so these pathological changes cannot currently be assessed directly with standard diagnostic tests.

MRI offers a unique opportunity to address this gap using quantitative tissue property mapping. T_1_ and T_2_ mapping are increasingly used in clinical practice for other organs, notably the heart, where T_1_ mapping is sensitive to fibrosis [15], infarction [16], inflammation [17], and infiltrative disease [18, 19], and T_2_ mapping reflects edema and acute inflammation [20, 21]. However, relatively few studies have explored quantitative mapping of the bladder wall. For instance, Tyagi et al. reported shorter post‐contrast bladder T_1_ values in patients with interstitial cystitis compared to healthy controls [22], Ye et al. applied radiomics to analyze T_2_ maps for staging of bladder cancer [23], and Yalcin et al. reported elevated native T_1_ values using a Modified Look‐Locker Inversion Recovery (MOLLI) sequence in patients with overactive bladder [24]. Despite these promising results, quantitative mapping in the bladder remains limited by technical challenges. The bladder wall is thin (3–5 mm in healthy adults), requiring high spatial resolution to reduce partial volume effects from urine and perivesical fat. Furthermore, conventional mapping requires a separate acquisition for each tissue property, which reduces scan efficiency and introduces potential co‐registration errors between maps.

Multiparametric techniques, such as magnetic resonance fingerprinting (MRF), offer a potential solution by simultaneously quantifying multiple tissue properties within a single scan [25]. MRF employs a specially designed time‐varying pulse sequence to generate unique signal evolutions dependent on underlying tissue properties. Signal evolutions (fingerprints) acquired during a highly accelerated scan are compared to a dictionary of simulated fingerprints, yielding co‐registered maps of multiple tissue properties. Recently, Correia et al. applied MRF to measure bladder wall T_1_, T_2_, and proton density (M_0_) in a small (*n* = 8) group of healthy female volunteers [26]. However, both this study and earlier single‐parametric approaches employed lower‐resolution 2D acquisitions (e.g., 1.6 × 1.6 mm^2^ in‐plane resolution with 3.5 mm slice thickness), resulting in limited coverage and greater susceptibility to partial volume artifacts.

The purpose of this study was to develop and evaluate a 3D MRF technique for simultaneous, co‐registered T_1_, T_2_, and M_0_ mapping of the entire bladder wall. The proposed method is based on a 3D stack‐of‐spirals MRF acquisition paired with a self‐supervised deep learning (deep image prior) reconstruction. A central aim was to characterize bladder wall thickness (BWT) and relaxation times using 3D MRF at both 1.5 T and 3 T in male and female subjects without known urologic disease across a broad age range, providing preliminary reference values and laying the groundwork for future studies using MRF to evaluate pathological bladder wall remodeling in LUTS.

## 2 Materials and Methods

### 2.1 3D Bladder MRF Pulse Sequence

**FIGURE 1:**
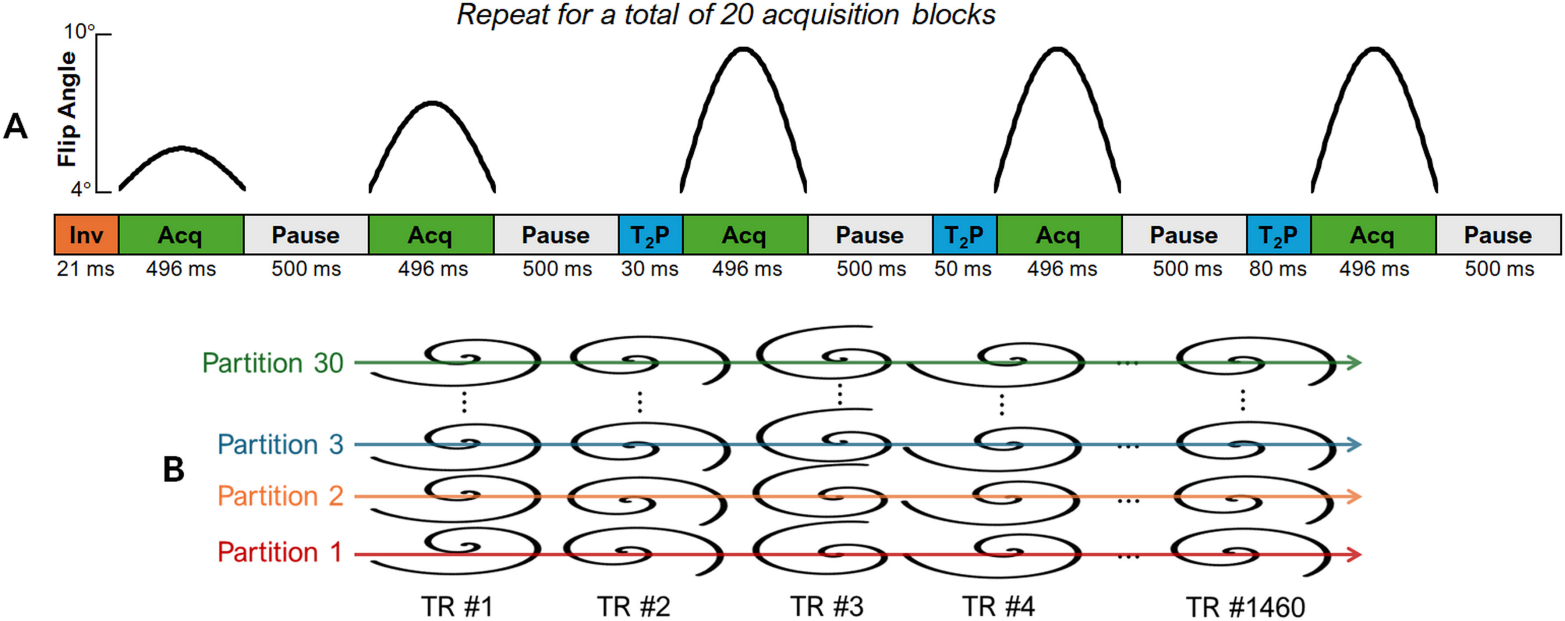
Schematic of the 3D bladder MRF sequence. (A) The pulse sequence consists of an RF‐spoiled gradient echo readout with sinusoidally varying flip angles (4°–10°), grouped into 20 acquisition blocks with 73 excitations. Each block is followed by a 500 ms recovery delay. Inversion (Inv) and T_2_ preparation (T_2_P) pulses are used to enhance sensitivity to T_1_ and T_2_. The diagram displays the first five acquisition blocks; this scheme is repeated four times for a total of 20 blocks and 1460 TRs. (B) Data are sampled using a 3D stack‐of‐spirals trajectory. A linear partition ordering is used, in which all MRF time points are acquired for one partition, followed by a 3‐s delay for partial T_1_ recovery before advancing to the next partition.

An MRF sequence was designed based on a 3D radiofrequency (RF)‐spoiled gradient echo sequence with small flip angles, selected to minimize sensitivity to B_0_ and B_1_^+^ inhomogeneities common in the pelvis (Figure 1). RF spoiling was achieved with a 117° RF phase increment and a spoiler gradient applied at the end of each repetition time (TR), producing 4 π dephasing per voxel along the partition axis. A total of 1460 TRs were collected, grouped into 20 acquisition blocks, each containing 73 excitations with sinusoidally varying flip angles between 4° and 10°. Each block was followed by a 500 ms pause to allow partial recovery of longitudinal magnetization. Excitation was performed using a 3D slab‐selective sinc pulse (1.6 ms duration, time‐bandwidth product 7.6). Preparation pulses were interleaved to enhance sensitivity to T_1_ and T_2_ [27]. The first acquisition block was preceded by an adiabatic inversion with an inversion time (TI) of 21 ms; the second block had no preparation; and the third, fourth, and fifth blocks were preceded by adiabatic BIR‐4 T_2_‐preparations [28] with durations of 30, 50, and 80 ms, respectively. This five‐block preparation scheme was repeated four times throughout the acquisition.

The k‐space data were sampled using a 3D stack‐of‐spirals trajectory. A uniform density spiral with 97 interleaves and 3.5 ms readout was designed [29] to cover a 300 × 300 mm^2^ field‐of‐view (FOV) on a 304 × 304 matrix, yielding an acquired voxel size of 1 × 1 × 3 mm^3^. The spiral was rotated between TRs using a pseudo‐golden‐angle scheme, in which the true golden angle increment was calculated, and the closest of the 97 available interleaves (equally spaced over 360°) was selected. A linear partition ordering was used, whereby all 1460 TRs were collected for one partition, followed by a 3‐s pause to allow partial T_1_ recovery and improve SNR before advancing to the next partition. The acquisition time was 23 s per partition. With 30 partitions for whole‐bladder coverage, the total scan time was 11.8 min.

### 2.2 MRF Reconstruction

A dictionary of fingerprints was precomputed using a Bloch equation simulation with 20,375 entries (T_1_ 50–4000 ms; T_2_ 5–500 ms). The dictionary incorporated corrections for imperfect inversion and T_2_ preparation efficiency [30]. Slice profile imperfections were not simulated because this was a 3D acquisition and uniform excitation was assumed across partitions. The dictionary was compressed using a singular value decomposition (SVD), retaining the top K=10 singular vectors [31]. The first K columns of the right singular matrix $\left(V_{K}\right)$ formed a low‐rank temporal basis. Projecting the MRF time‐series data onto this basis (by multiplying with $V_{K}$) compressed the data along the time dimension, yielding K subspace images.

Before reconstruction, the k‐space data were compressed to 8 virtual coils using the region‐optimized virtual coils (ROVir) method, placing the signal region within the supported 300 × 300 mm^2^ FOV and the interference region in the outer 2‐fold oversampled area [32]. A 1D FFT was then applied along the partition direction, allowing each slice to be reconstructed independently using a 2D deep image prior (DIP) reconstruction. DIP is a self‐supervised deep learning method that requires no external training data, with training performed *de novo* for each scan using only the acquired undersampled k‐space data [33, 34]. Details about the DIP reconstruction are provided in Supporting Figure S1. Briefly, a fixed random code tensor was input to a randomly initialized U‐Net, which generated the subspace images. This U‐Net was optimized with a loss function that enforced consistency with the measured k‐space data through a forward encoding model, including low‐rank subspace projection and spiral sampling. Concurrently, a multilayer perceptron was trained to estimate quantitative maps from the subspace images. Independent DIP reconstructions were performed in parallel for all slices on a high‐performance cluster, reserving one GPU with 16 GB memory per slice. Training was performed for 300 epochs using an Adam optimizer, a learning rate of 0.001, and 5% dropout. Each slice required approximately 2 h to reconstruct, with multiple slices processed in parallel on different nodes to reduce the overall computation time for the 3D volume.

In one subject, the same MRF dataset was reconstructed using three approaches to evaluate the effect of reconstruction method: (1) direct matching, in which undersampled images were reconstructed with a non‐uniform fast Fourier Transform (NUFFT) [35] and matched to the dictionary, as in the original MRF framework [25]; (2) an iterative sparse and locally low‐rank (SLLR) reconstruction, which uses a temporal subspace derived from the MRF dictionary together with total variation and locally low‐rank regularization [36]; and (3) the DIP reconstruction.

### 2.3 Synthetic Weighted Images

Qualitative weighted images were generated from the MRF maps to measure BWT. First, T_2_ and M_0_ maps were used to calculate T_2_‐weighted spin echo images with adjustable echo time (TE).

(Eq 1) $$S_{T2w}\left(\mathrm{TE}\right)=M_{0}e^{-\frac{\mathrm{TE}}{T_{2}}}$$

Second, T_1_ and M_0_ maps were used to calculate T_1_‐weighted phase‐sensitive inversion recovery (PSIR) images with adjustable TI.

(Eq 2) $$S_{\text{PSIR}}\left(\mathrm{TI}\right)=M_{0}\left(1-2e^{-\frac{\mathrm{TI}}{T_{1}}}\right)$$

A long TI was selected to generate a FLAIR‐like (black‐urine) contrast, nulling signal from urine to improve bladder wall delineation. Assuming a urine T_1_ of approximately 3600 ms (based on MRF measurements), the optimal TI was calculated as 2500 ms.

(Eq 3) $$\mathrm{TI}=T_{1}^{\text{urine}}\cdot \ln\left(2\right)$$

### 2.4 Study Population and MRI Protocol

Data were collected from 29 healthy participants without known urologic disease (14 female; age range 18–78 years; median 41 years, interquartile range 26–51 years) after obtaining written informed consent in this IRB‐approved, HIPAA‐compliant study. Validated LUTS questionnaires and functional urologic testing were not performed, so subclinical lower urinary tract dysfunction (LUTD) could not be excluded. Within this group, 16 subjects underwent two consecutive 30‐min MRI exams on 1.5 T (MAGNETOM Sola) and 3 T scanners (MAGNETOM Vida, Siemens Healthineers, Erlangen, Germany), with exam order randomized for each participant. The remaining subjects were scanned at only one field strength due to scheduling constraints (*n* = 11 at 1.5 T only, *n* = 2 at 3 T only). Subjects voided immediately before the exam at each field strength to minimize interscan variability in bladder volume and increase bladder wall thickness, thereby reducing partial volume effects in the maps. At 1.5 T, imaging was performed using an 18‐channel body array coil combined with 10–14 spine array channels. At 3 T, imaging used a 30‐channel body array coil and 10–14 spine array channels. All scans were acquired in a sagittal orientation.

At both field strengths, 3D MRF scans used identical sequence parameters (300 × 300 × 90 mm^3^ FOV; scan time 11.8 min). MRF data were acquired with a voxel size of 1 × 1 × 3 mm^3^, with zero‐filling interpolation to an apparent resolution of 0.5 × 0.5 × 1.5 mm^3^. Because standardized clinical sequences for quantitative bladder mapping do not exist, clinical cardiac mapping sequences were adapted for higher spatial resolution (0.8 × 0.8 mm^2^ in‐plane resolution after interpolation; 5 mm slice thickness) following the approach described by Yalcin et al. [24] These were used to acquire conventional 2D T_1_ and T_2_ maps from a mid‐sagittal slice for comparison with 3D MRF. T_1_ maps were collected using MOLLI [37], and T_2_ mapping was performed with a T_2_‐prepared gradient echo (GRE) sequence [20]. These scans employed a balanced steady‐state free precession (bSSFP) readout at 1.5 T to improve SNR and an RF‐spoiled readout at 3 T to minimize off‐resonance artifacts. Conventional mapping scans were not acquired in 3 of 27 exams at 1.5 T and 4 of 17 exams at 3 T because of time constraints. In addition, conventional T_2_‐weighted images were collected using a 2D multislice fast spin echo (FSE) sequence (0.5 × 0.5 mm^2^ in‐plane resolution after interpolation; 3 mm slice thickness) to provide reference measurements of BWT. Additional scan parameters are listed in Supporting Table S1.

### 2.5 Image Analysis

Maps were analyzed by placing regions of interest (ROIs) in the bladder wall using ITK‐SNAP [38]. Because the MRF T_1_ and T_2_ maps were inherently co‐registered, they were viewed together when drawing ROIs. First, a midline sagittal slice was selected by identifying the cartilaginous pubic symphysis and bladder neck as landmarks. Global mean and standard deviation (SD) values for T_1_ and T_2_ were calculated over all bladder wall voxels within this midline slice. Next, regional T_1_ and T_2_ values were measured by drawing ROIs in the following midline locations (Supporting Figure S2): (1) trigone, segmented from the posterior bladder neck to the ureteral orifices; (2) base, segmented from the anterior bladder neck along the horizontal plane and terminating before the vertical aspect of the bladder; (3) anterior wall, segmented along the vertical aspect of the bladder wall; (4) dome, segmented along the superior horizontal aspect of the bladder; and (5) posterior wall, segmented along the posterior curvature of the bladder superior to the trigone, terminating at the dome. Lateral walls were segmented approximately 20 mm to the (6) left and (7) right of the midline slice, peripheral to and aligned with the trigone. These segmentations were used to assess whether the trigone—a highly innervated area that signals the need to void—has distinct T_1_ and T_2_ characteristics relative to other bladder wall regions. To ensure sufficient voxel counts for reliable T_1_ and T_2_ measurements, all ROIs were extended to two adjacent slices (one on each side of the original slice). Voxels affected by motion or partial volume averaging with urine or perivesical fat were manually avoided.

Mean T_1_ and T_2_ values were computed within each region. Variability was quantified using two metrics. First, variability across subjects was quantified using the intersubject coefficient of variation (CV), defined as the SD of the mean T_1_ or T_2_ within each ROI normalized by the overall mean across subjects. Second, the spatial heterogeneity of bladder wall T_1_ and T_2_ measurements was quantified using the spatial CV, computed as the SD of voxel values within each ROI (normalized by the mean within the ROI) and then averaged across subjects.

Synthetic weighted images generated from MRF maps were used to measure BWT and were validated against reference values from conventional T_2_‐weighted images. BWT values were measured regionally in the trigone, base, anterior wall, dome, and posterior wall at the midline slice. Within each region, three line profiles were drawn perpendicular to the lumen at the point of maximal wall thickness, and their average was recorded. Global BWT was then computed as the average over all regions. Synthetic T_1_‐weighted PSIR and T_2_‐weighted images from MRF were viewed together to improve wall delineation when measuring BWT.

### 2.6 Statistical Analysis

Agreement between T_1_ and T_2_ measurements from 3D MRF and 2D mapping sequences, as well as BWT from synthetic and conventional T_2_‐weighted images, was assessed using paired *t*‐tests and Bland–Altman analyses [39]. Comparisons between 1.5 T and 3 T measurements were performed using paired *t*‐tests in the subset of 16 subjects scanned at both field strengths. Regional differences in relaxation times and BWT were evaluated separately at each field strength with one‐way repeated‐measures ANOVA, followed by post hoc paired *t*‐tests with Bonferroni correction. Sex differences in regional T_1_, T_2_, and BWT were assessed with independent samples *t*‐tests, with Bonferroni correction for the multiple bladder wall regions. Associations between age and T_1_, T_2_, and BWT were assessed with linear regression, reporting the Pearson correlation coefficient, regression slope, and two‐sided *p* value. Given the modest sample size, age‐ and sex‐related analyses were treated as exploratory and were not intended to establish age‐ or sex‐stratified normative ranges. Statistical significance was defined as *p* < 0.05, after correction where applicable.

## 3 Results

### 3.1 Representative Maps and Synthetic Images

**FIGURE 2:**
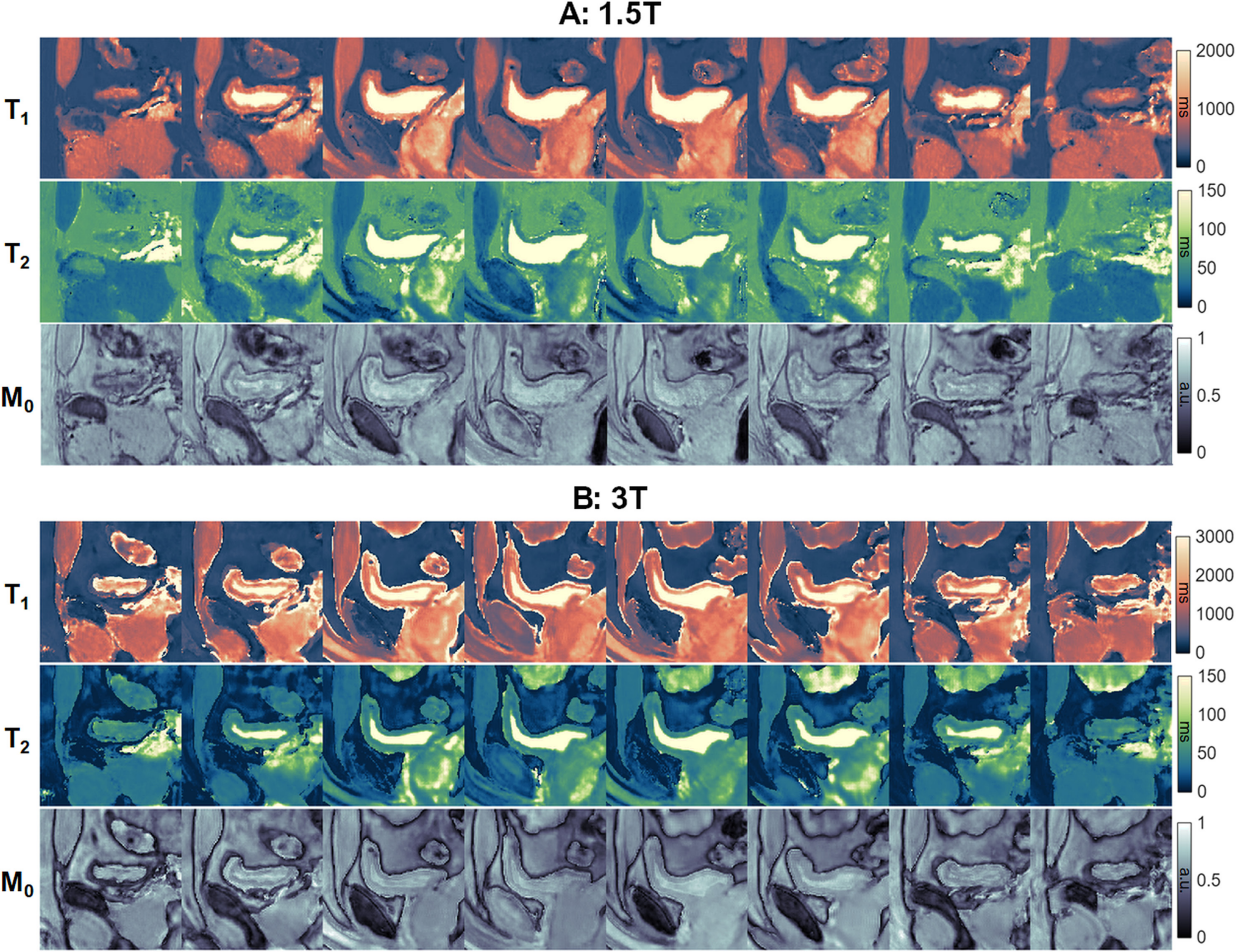
Representative 3D MRF T_1_, T_2_, and M_0_ maps from a 41‐year‐old male subject at (A) 1.5 T and (B) 3 T. Maps are displayed in sagittal orientation, with multiple partitions shown from left to right. Note the difference in scale bars for T_1_ maps at each field strength. Maps were cropped to a 100 × 100 region centered on the bladder. Full FOV maps are provided in Supporting Figure S3.

**FIGURE 3:**
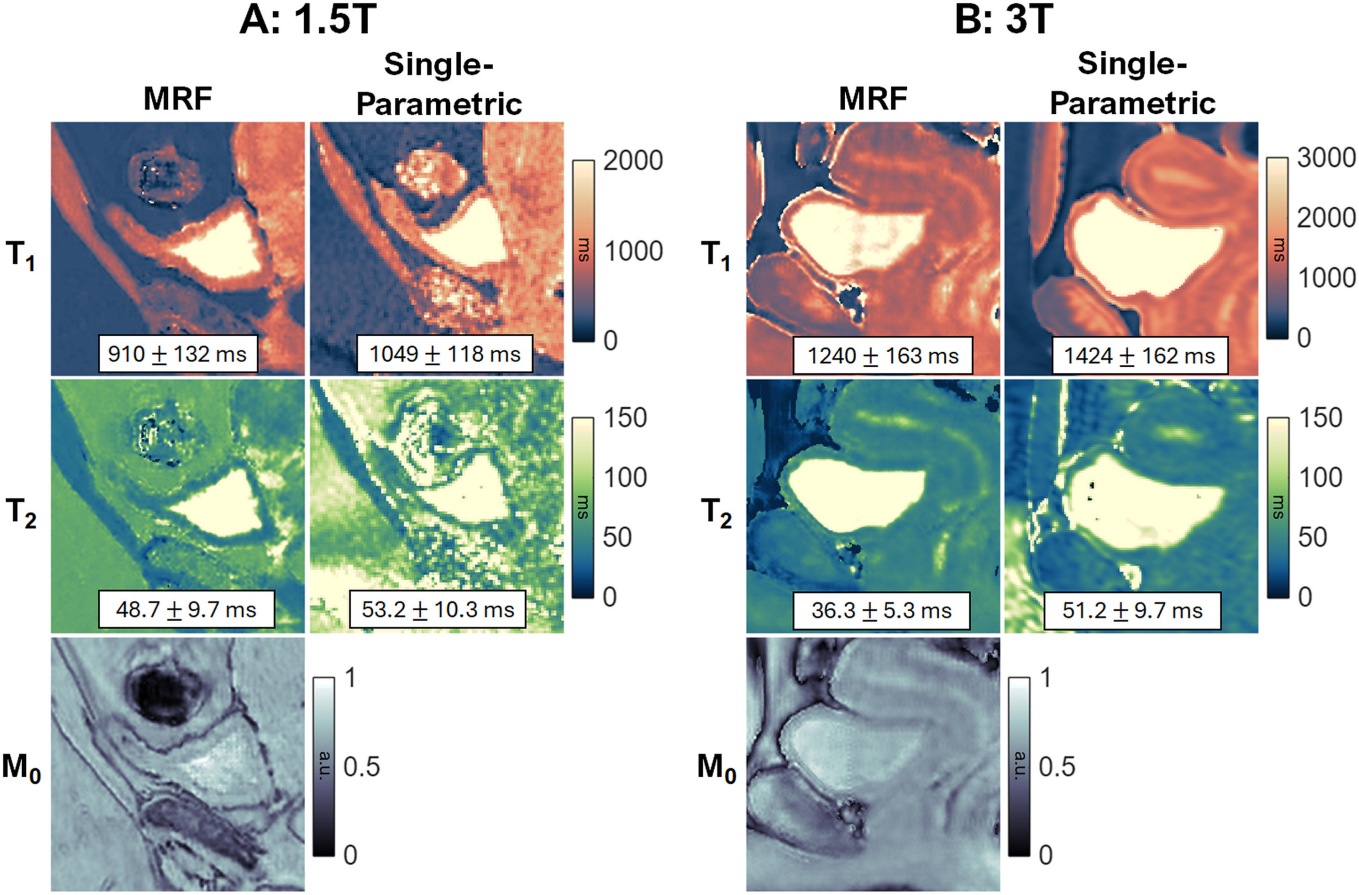
Comparison of 3D MRF and 2D single‐parametric mapping sequences from a mid‐sagittal slice of the bladder. (A) Maps from a 33‐year‐old female at 1.5 T. (B) Maps from a 20‐year‐old female at 3 T. Global bladder wall T_1_ and T_2_ values are displayed in the insets as mean ± SD.

Representative co‐registered 3D T_1_, T_2_, and M_0_ maps acquired with MRF in one subject at both 1.5 T and 3 T are displayed in Figure 2 and Supporting Figure S3. The maps demonstrated clear delineation of the bladder wall from the lumen and surrounding tissues, with visual uniformity within the wall. Maps from three additional subjects are shown in Supporting Figures S4–S6. Figure 3 compares 3D MRF with lower‐resolution 2D single‐parametric mapping sequences at both field strengths. Qualitatively, the single‐parametric maps were noisier than the MRF maps at 1.5 T. These differences were less pronounced at 3 T. In this subject, a focal region with elevated T_2_ was observed in the anterior wall on the single‐parametric T_2_ map at 3 T but was absent in the higher‐resolution 3D MRF map, consistent with partial volume averaging with urine in the conventional acquisition.

**FIGURE 4:**
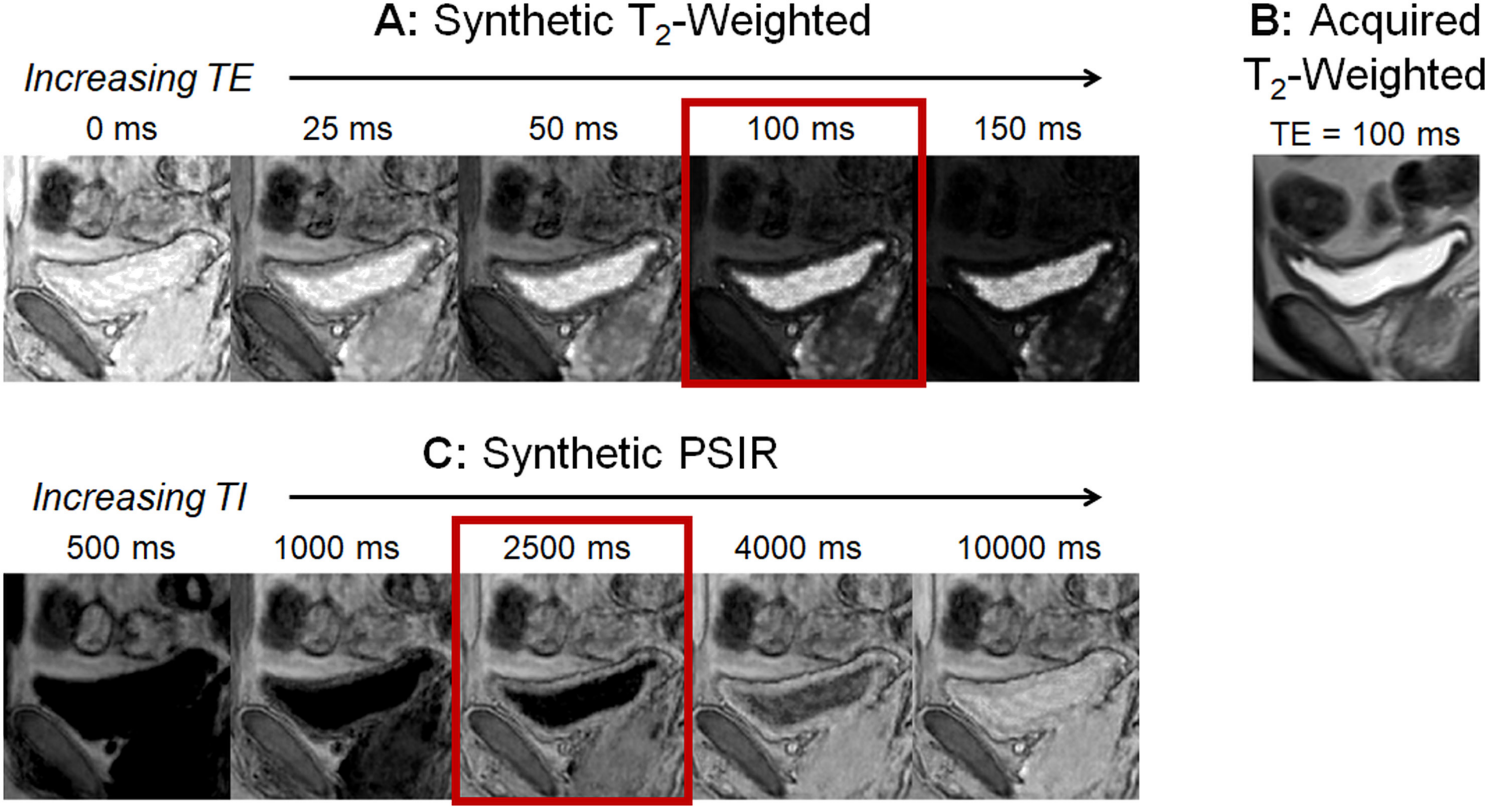
Synthetic images derived from 3D MRF maps at 1.5 T. (A) Synthetic T_2_‐weighted images are shown with echo times ranging from 0 to 150 ms, alongside (B) a conventional T_2_‐weighted FSE image with an effective echo time of 100 ms. (C) Synthetic T_1_‐weighted PSIR images are shown with inversion times ranging from 500 ms to 10 s. The synthetic images used for BWT measurement are highlighted, corresponding to TE = 100 ms for the T_2_‐weighted image and TI = 2500 ms for the PSIR image.

Synthetic weighted images calculated from MRF maps are presented in Figure 4, which displays T_2_‐weighted images across a range of TEs and T_1_‐weighted PSIR images across a range of TIs, alongside a conventional T_2_‐weighted image. When the synthetic TE was matched to the conventional T_2_‐weighted scan (TE = 100 ms), the resulting image showed hypointense signal in bladder wall and hyperintense signal in urine, similar in appearance to the conventional image. Synthetic PSIR images with a long TI (e.g., 2500 ms) produced a black‐urine contrast intended to improve conspicuity of the bladder wall.

**FIGURE 5:**
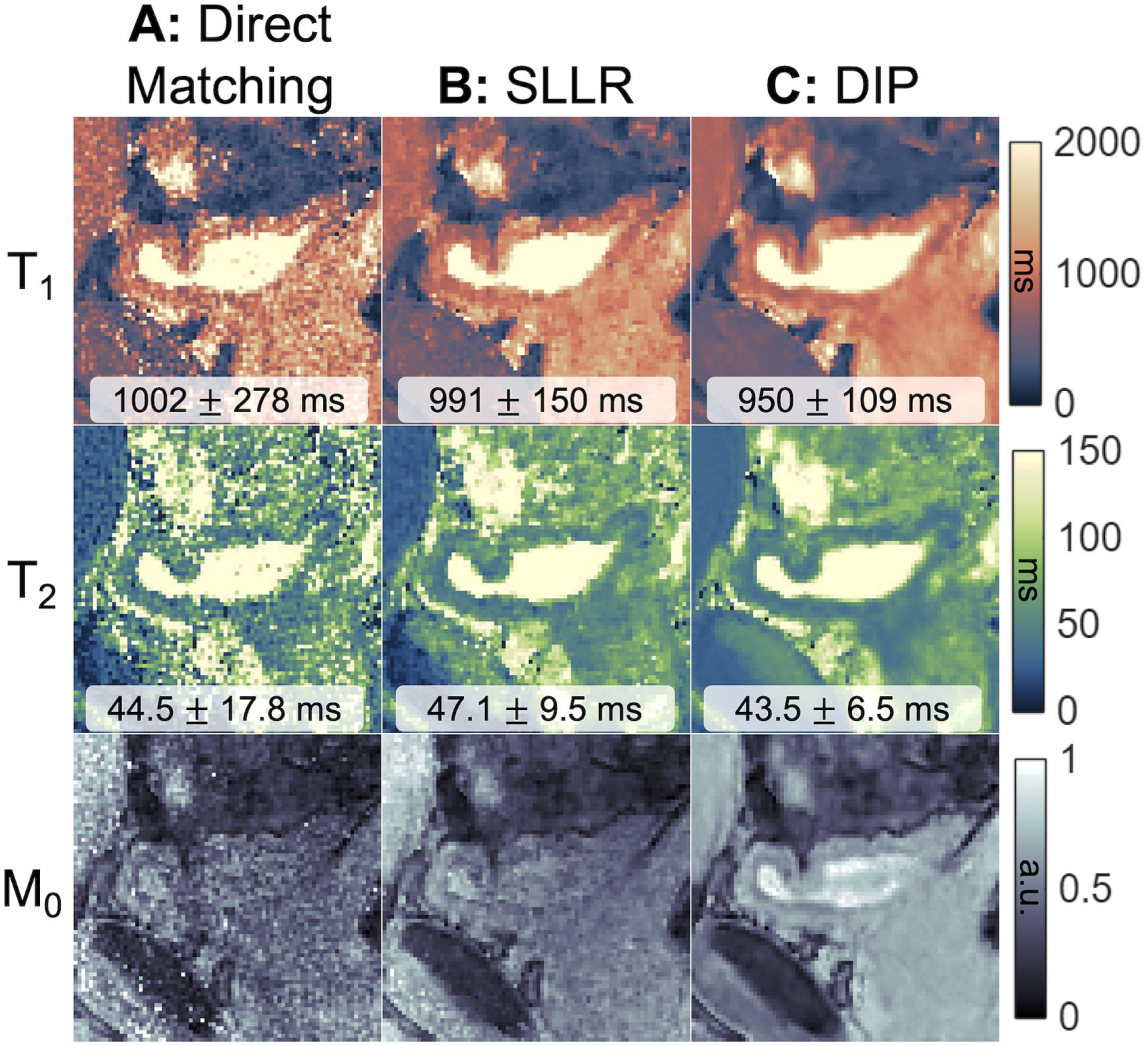
Comparison of MRF reconstruction methods. The same MRF k‐space data from one representative subject at 1.5 T were reconstructed using three different approaches: (A) direct matching, (B) SLLR, and (C) DIP reconstructions. The mean and standard deviation of global bladder wall relaxation times are reported on the T_1_ and T_2_ maps.

The effect of reconstruction method on map quality is shown in Figure 5 for direct matching, SLLR, and DIP reconstructions of the same dataset. Mean bladder wall T_1_ and T_2_ values were similar across methods, whereas the standard deviations within the wall decreased progressively from direct matching to SLLR to DIP. DIP provided the clearest delineation of the bladder wall with improved noise suppression. Zero‐filling interpolation (Supporting Figure S7) further improved the appearance of the maps, producing a smoother, less pixelated depiction of the bladder wall without affecting the tissue property estimates.

### 3.2 Global and Regional Bladder Wall Measurements

**FIGURE 6:**
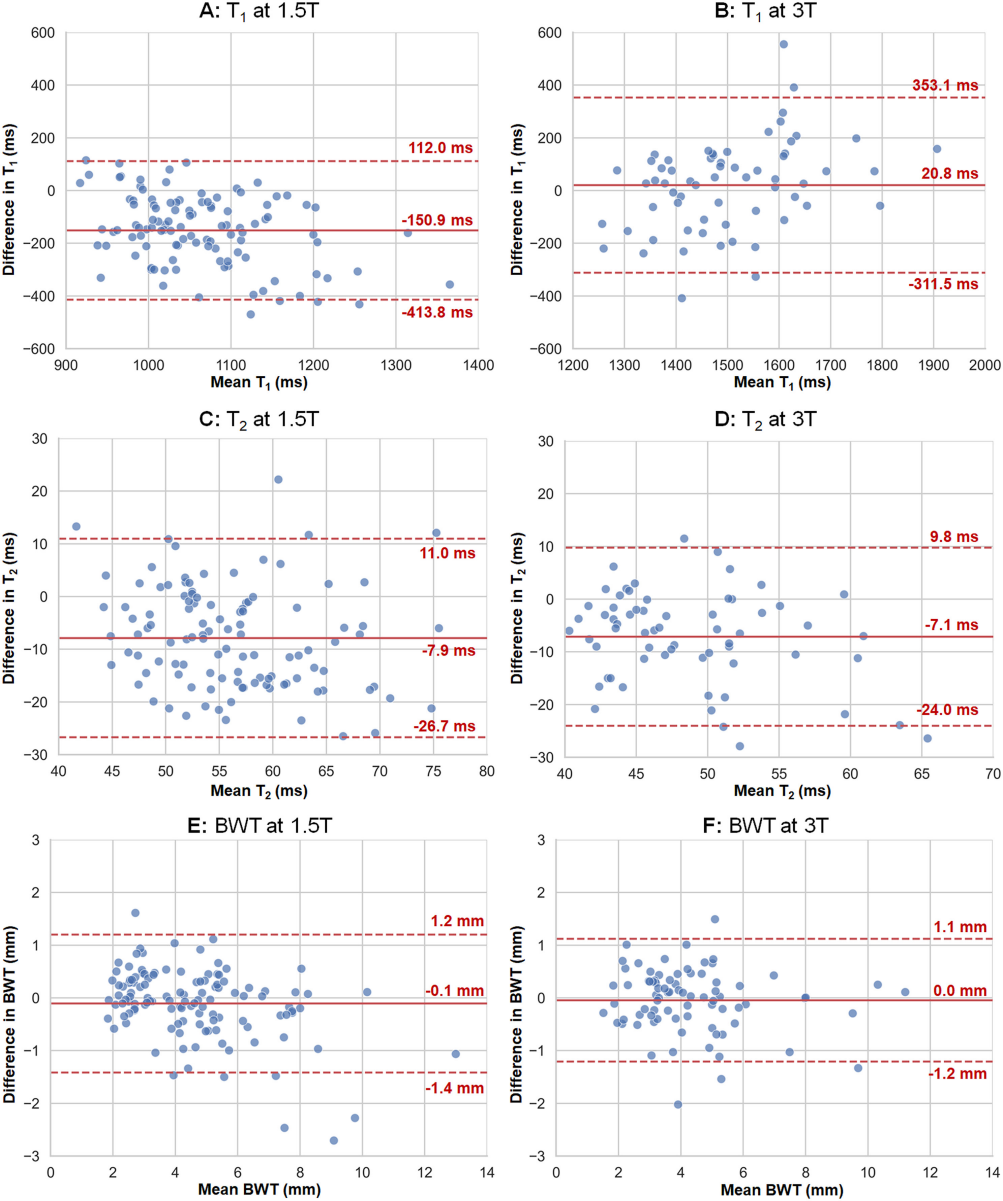
Agreement between MRF and single‐parametric mapping. Bland–Altman plots are shown comparing (A,B) T_1_ values from MRF and MOLLI, (C,D) T_2_ values from MRF and T_2_‐prepared GRE mapping, and (E,F) BWT values from synthetic MRF‐derived images and conventional T_2_‐weighted imaging at both 1.5 T and 3 T. Data points represent the mean T_1_, T_2_, or BWT value measured within a single bladder segment for each subject. The mean bias is indicated by a solid red line, with the 95% upper and lower limits of agreement shown as dotted red lines.

Global bladder wall relaxation times and BWT are summarized in Table 1, reported as mean ± intersubject SD. Consistent with the expected field strength dependence, mean T_1_ values were significantly higher at 3 T than at 1.5 T, while T_2_ values were significantly lower at 3 T for both MRF and single‐parametric mapping (*p* < 0.001). Mean global MRF T_1_ increased from 984 ± 53 ms (1.5 T) to 1468 ± 118 ms (3 T), while T_2_ decreased from 51.4 ± 5.6 to 43.5 ± 4.0 ms. Compared to 2D single‐parametric mapping sequences, MRF T_1_ values were significantly lower than MOLLI at 1.5 T (*p* < 0.001), with a bias of −151 ms on a Bland–Altman analysis (Figure 6); however, no significant difference was observed at 3 T (mean bias 21 ms). MRF T_2_ values were significantly lower than T_2_‐prepared GRE mapping at both field strengths (*p* < 0.001), with mean biases of −8 ms at 1.5 T and −7 ms at 3 T. As expected, BWT did not differ significantly between field strengths, with mean global values of 4.5 ± 1.6 mm at 1.5 T and 4.3 ± 1.6 mm at 3 T on synthetic MRF‐derived images. A Bland–Altman analysis revealed negligible bias relative to conventional T_2_‐weighted images (−0.1 mm at 1.5 T and 0.0 mm at 3 T).

**TABLE 1:** Global bladder wall measurements. Global bladder wall T_1_, T_2_, and BWT at 1.5 T and 3 T using 3D MRF and 2D single‐parametric mapping sequences, reported as mean ± SD.

Field strength	Scan	Global T_1_ (ms)	Global T_2_ (ms)	Global BWT (mm)
**1.5 T**	**3D MRF**	984 ± 53	51.4 ± 5.6	4.5 ± 1.6
	**2D single‐parametric**	1129 ± 85	60.2 ± 6.2	4.3 ± 1.6
**3 T**	**3D MRF**	1468 ± 118	43.5 ± 4.0	4.3 ± 1.6
	**2D single‐parametric**	1487 ± 50	51.5 ± 5.4	4.6 ± 1.8

**FIGURE 7:**
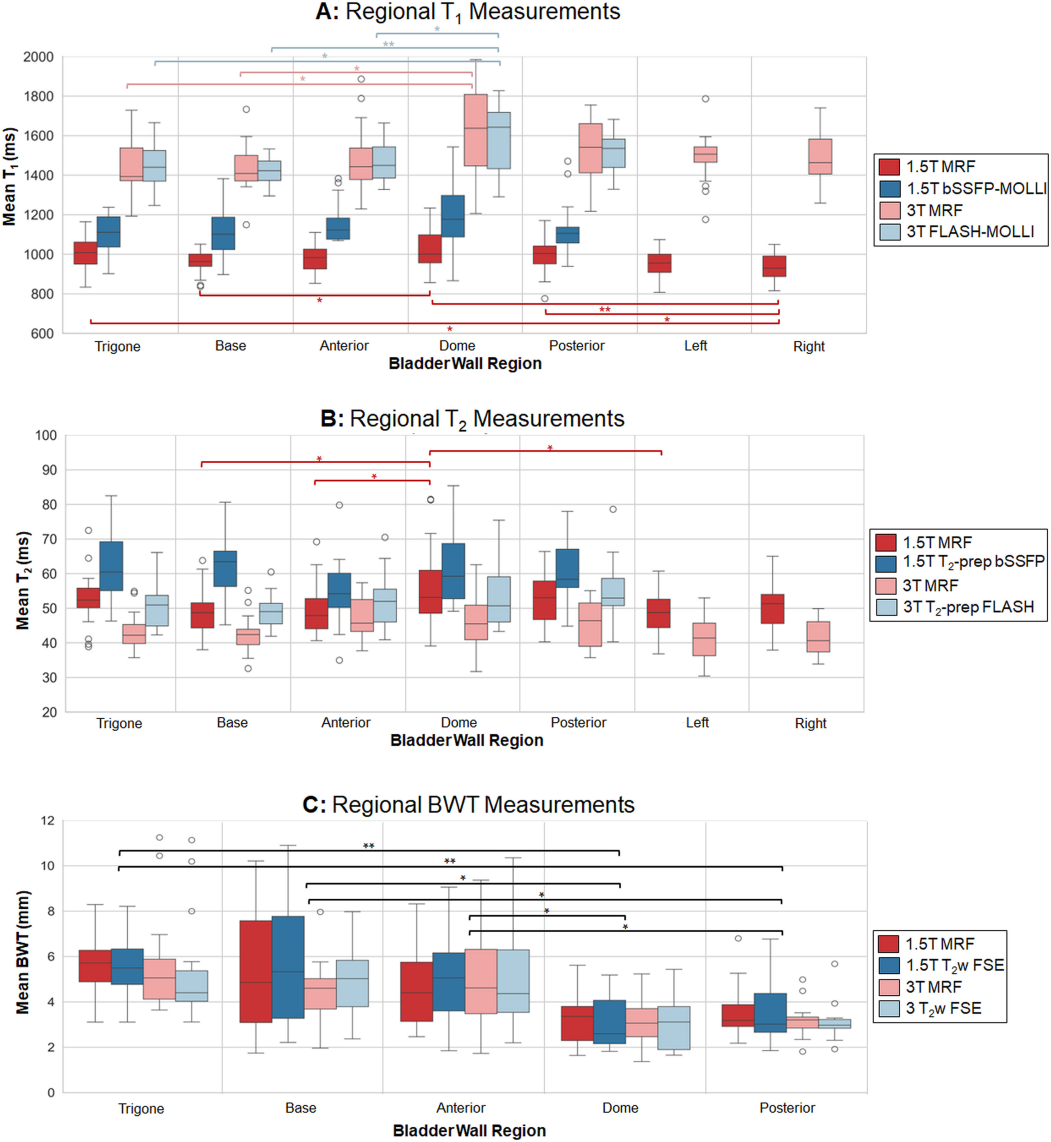
T_1_, T_2_, and BWT measurements in different bladder wall regions. The distribution of mean (A) T_1_, (B) T_2_, and (C) BWT values is shown across bladder wall segments measured at both 1.5 T and 3 T using MRF and comparison sequences. For T_1_ and T_2_ (A,B), significant regional differences at each field strength and for each acquisition method are marked with color‐coded asterisks (*p < 0.05, **p < 0.01, ***p < 0.001). For BWT (C), significance is indicated with a single marker, rather than separate markers for each field strength and method, since all methods showed consistent results.

Regional bladder wall T_1_ and T_2_ measurements are summarized in Figure 7 and Supporting Tables S2 and S3. MRF relaxation times were generally comparable across the trigone, base, anterior, and lateral regions, whereas the dome and posterior wall exhibited higher mean T_1_ and T_2_ values. For example, mean MRF values (T_1_, T_2_) at 1.5 T were (1024, 56) ms in the dome compared to (962, 49) ms in the base; at 3 T, they were (1624, 46) ms in the dome and (1435, 43) ms in the base. Overall, no significant differences were found between the trigone and other regions, except for a slightly higher mean T_1_ compared to the right lateral wall at 1.5 T (1008 vs. 935 ms, *p* < 0.05). A gradient in BWT was observed, with the bladder wall significantly thicker (4.4–5.9 mm) in the trigone, base, and anterior regions and thinner (3.2–3.5 mm) in the dome and posterior regions, which are more distensible (Supporting Table S4).

### 3.3 Measurement Variability

Variability in global bladder wall MRF measurements across subjects was comparable at both field strengths, with intersubject CV values (T_1_, T_2_) of (8.1%, 14.7%) at 1.5 T and (10.9%, 14.2%) at 3 T. Intersubject CV values for single‐parametric 2D mapping scans were (12.0%, 9.2%) at 1.5 T and (7.5%, 7.9%) at 3 T. Variability in global BWT across subjects was consistent across field strengths and methods, with intersubject SD values of 1.6 mm (1.5 T MRF), 1.6 mm (3 T MRF), 1.8 mm (1.5 T conventional T_2_‐weighted images), and 1.6 mm (3 T conventional T_2_‐weighted images). Intersubject variability for T_1_, T_2_, and BWT within each bladder wall region is shown in Supporting Figure S8; although no significant regional differences were observed, the dome generally showed higher intersubject variability in T_1_ and T_2_.

Spatial heterogeneity was quantified using the spatial CV of global bladder wall T_1_ and T_2_ measurements, a metric of precision (Supporting Figure S9). For MRF, no significant differences were observed between 1.5 T (T_1_ CV 13.9%, T_2_ CV 20.9%) and 3 T scans (T_1_ CV 14.5%, T_2_ CV 19.9%). At 1.5 T, MRF yielded significantly improved T_1_ precision compared to MOLLI (CV 16.0%, *p* < 0.05), with a similar nonsignificant trend relative to T_2_‐prepared GRE (CV 23.0%). Precision was also analyzed within each bladder wall region (Supporting Figure S10) and was generally improved (lower CV) in the trigone, base, and anterior wall than in the dome and posterior wall. For example, spatial CV values with MRF in the dome were 14.7% (T_1_)/20.1% (T_2_) at 1.5 T and 13.2% (T_1_)/18.8% (T_2_) at 3 T, compared to 9.6% (T_1_)/16.1% (T_2_) at 1.5 T and 9.5% (T_1_)/14.2% (T_2_) at 3 T in the trigone.

### 3.4 Exploration of Age and Sex Associations

**FIGURE 8:**
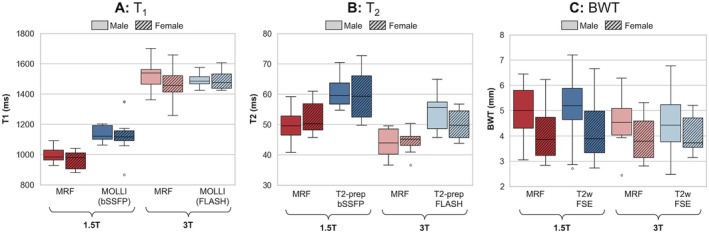
Sex differences in global bladder wall measurements. Global measurements of (A) T_1_, (B) T_2_, and (C) BWT are presented for male and female subjects using MRF and conventional MRI sequences at both 1.5 T and 3 T.

Male and female subjects showed no significant differences in global bladder wall relaxation times or BWT, although BWT tended to be higher in men on both synthetic MRF‐derived images and conventional T_2_‐weighted images at both field strengths (Figure 8). With MRF, global mean T_1_ values were 1000 ± 46 ms (men) and 967 ± 55 ms (women) at 1.5 T, and 1512 ± 114 ms (men) and 1457 ± 117 ms (women) at 3 T; global mean T_2_ values were 50.1 ± 5.7 ms (men) and 52.7 ± 5.3 ms (women) at 1.5 T, and 42.8 ± 3.8 ms (men) and 44.2 ± 4.0 ms (women) at 3 T; and global mean BWT values were 5.0 ± 1.2 mm (men) and 4.1 ± 1.1 mm (women) at 1.5 T, and 4.5 ± 1.1 mm (men) and 4.0 ± 0.8 mm (women) at 3 T. Comparisons of T_1_, T_2_, and BWT within different bladder wall regions between men and women are shown in Supporting Figure S11. In the trigone, mean T_1_ was higher in men than women at both field strengths (1058 ± 73 ms vs. 957 ± 62 ms at 1.5 T, and 1496 ± 153 ms vs. 1392 ± 113 ms at 3 T); the difference was statistically significant at 1.5 T (*p* < 0.05). Trigone T_2_ values also tended to be higher in men than women (54.9 ± 8.0 vs. 50.2 ± 5.5 ms at 1.5 T, and 45.1 ± 6.4 vs. 41.6 ± 3.3 ms at 3 T). BWT values were consistently higher in men across all regions, although only significant in the dome at 3 T (*p* < 0.05).

**FIGURE 9:**
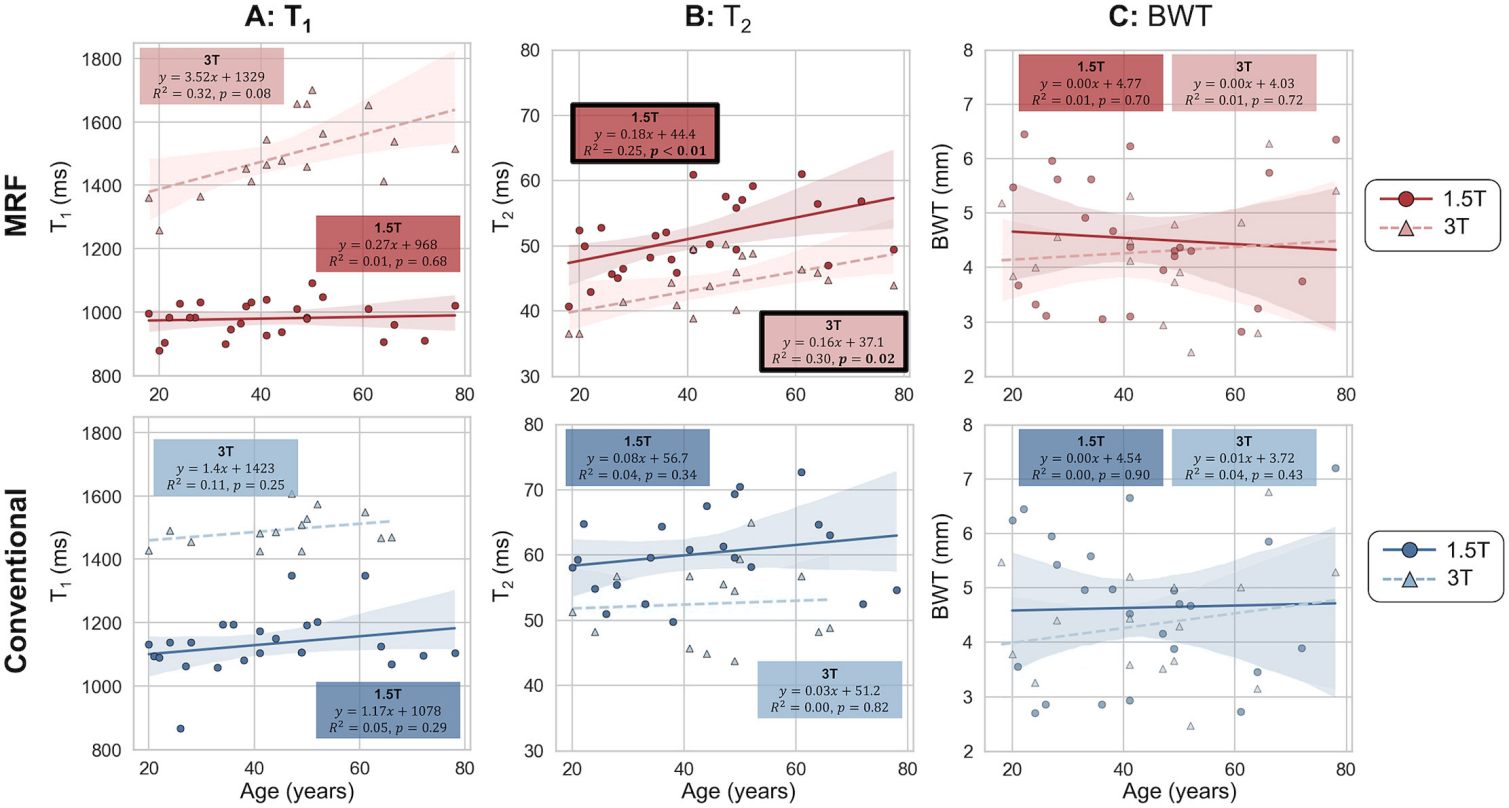
Association between bladder wall relaxation times and thickness with age. Global (A) T_1_, (B) T_2_, and (C) BWT measurements are shown as a function of age for MRF (top row) and conventional MRI sequences (bottom row) at 1.5 T and 3 T. Best‐fit regression lines are reported, along with coefficients of determination (*R* ^2^ values) and *p* values. The shaded areas indicate the 95% confidence interval for the regression. Conventional mapping data were unavailable in the two oldest subjects at 3 T.

In an exploratory analysis, global MRF T_2_ values showed a weak positive association with age at both field strengths (Figure 9), although this finding may be confounded by unrecognized age‐related LUTD in older participants. Conventional T_2_ mapping showed no significant association with age. No significant age‐related associations were observed for global T_1_ or BWT with either MRF or conventional sequences. Regression plots comparing age with regional MRF measurements are provided in Supporting Figure S12. For T_1_, weak positive associations with age were observed in all regions but only at 3 T, reaching statistical significance in the trigone and posterior wall (*p* < 0.05). Positive associations between T_2_ and age were observed in all regions at both field strengths, reaching significance in the anterior and posterior wall at 1.5 T (*p* < 0.01) and in the trigone and posterior wall at 3 T (*p* < 0.05). A relationship between BWT and age was observed only in the posterior wall, where a significant negative association was observed at both field strengths (*p* < 0.05).

## 4 Discussion

This study introduced a novel 3D MRF approach for simultaneous T_1_, T_2_, and M_0_ mapping of the bladder wall, combining an undersampled stack‐of‐spirals acquisition with a deep image prior reconstruction. Whole‐bladder 3D maps were acquired at a spatial resolution of 1 × 1 × 3 mm^3^ (interpolated to 0.5 × 0.5 × 1.5 mm^3^) in 11.8 min. Unlike conventional mapping approaches that measure a single tissue property from a relatively thick 2D slice, 3D MRF provides whole‐bladder multiparametric maps and synthetic weighted images that are inherently co‐registered because they are derived from a single acquisition. Compared with prior 2D bladder mapping studies [26], 3D MRF also provides finer resolution, particularly along the slice direction, which may aid delineation of the thin bladder wall. Bladder wall relaxation times were characterized in healthy subjects scanned in back‐to‐back exams at 1.5 T and 3 T, and BWT was quantified from synthetic T_1_‐ and T_2_‐weighted images derived from the MRF maps with no additional scan time. To our knowledge, this represents the first application of 3D MRF for quantitative bladder wall tissue characterization at both 1.5 T and 3 T.

Routine methods for functional and structural assessment of LUTS do not capture the microstructural tissue remodeling associated with functional urologic conditions. Quantitative imaging using MRF offers a novel avenue to characterize these underlying tissue properties. Establishing feasibility and characterizing bladder MRF measurements at both 1.5 T and 3 T are essential first steps toward clinical translation. In this study, the same MRF protocol was successfully deployed at both field strengths. Interestingly, comparable T_1_ and T_2_ mapping precision was observed at 1.5 T and 3 T. Although 3 T provides inherently higher SNR, it suffers from increased B_0_ and B_1_^+^ inhomogeneities that can introduce image artifacts or quantification errors. These competing factors may have counterbalanced each other, leading to similar precision at each field strength. This study also employed an RF‐spoiled MRF sequence with small flip angles chosen for its low sensitivity to B_0_ and B_1_^+^ variations, enabling the same scan to be deployed at both field strengths without B_0_ or B_1_^+^ correction.

MRF has the potential to provide non‐invasive, quantitative information about bladder tissue composition that is not captured by standard functional assessments (uroflowmetry and UDS) or anatomic imaging (ultrasound, CT, conventional MRI) for LUTS. By quantifying bladder wall alterations such as fibrosis, inflammation, and edema, this approach could extend to the bladder the diagnostic value that T_1_ and T_2_ mapping has shown in cardiac MRI [37]. Potential clinical applications include assessing pathological remodeling in bladder outlet obstruction, overactive and neurogenic bladder, monitoring chronic inflammatory conditions such as interstitial cystitis, and quantitative staging of bladder malignancies.

### 4.1 Comparison With Prior Work

Relaxation times were overall consistent with prior literature. For example, anterior bladder wall values at 3 T measured with the proposed RF‐spoiled 3D MRF sequence (T_1_ 1468 ± 182 ms; T_2_ 52 ± 9 ms) closely matched those reported by Correia et al. using a lower‐resolution 2D FISP MRF sequence (T_1_ 1476 ± 138 ms; T_2_ 53 ± 8 ms) [26]. The 3D MRF T_1_ values measured in the anterior wall (973 ± 68 ms) agreed with measurements by Yalcin et al. using MOLLI (989 ± 81 ms) at 1.5 T [24]; however, they were lower than the 1.5 T MOLLI measurements obtained in the current study (1132 ± 164 ms). At both field strengths, MRF T_2_ values were 7–8 ms lower on average compared to single‐parametric mapping. This systematic bias is consistent with MRF studies in other organs and may be attributed to several factors, including magnetization transfer [40] and intravoxel dephasing [41]. While MOLLI and T_2_‐prepared GRE mapping were included for comparison, these are not established reference standards for the bladder, as they were modified from clinical cardiac protocols. Moreover, these techniques are sensitive to many confounding factors [42], and they employed 2D acquisitions with lower spatial resolution, particularly along the slice direction, which may increase susceptibility to partial volume artifacts.

### 4.2 Regional Variation

Unlike cardiac MRI, where T_1_ and T_2_ maps are segmented using the standardized American Heart Association model [43], no established framework exists for the bladder, so a custom segmentation approach was adapted from a previously described model [44]. T_1_ and T_2_ values were slightly higher in the bladder dome and posterior wall compared to the base and anterior walls. These differences may partly reflect motion (e.g., respiratory motion, bowel peristalsis) or passive bladder filling during the scan, as the dome is highly distensible [45]. Partial volume averaging is also likely, as these regions had the thinnest BWT, making them more susceptible to signal contamination from urine and perivesical fat; regional measurements in the dome and posterior wall should therefore be interpreted with caution. The trigone was also analyzed as a distinct region of interest because it is highly innervated with stretch receptors that signal the urge to void and has reduced smooth muscle content. However, trigone relaxation times were found to be comparable to other bladder wall regions.

### 4.3 Exploration of Age and Sex Associations

Age‐ and sex‐dependent differences in relaxation times and BWT were explored in this study, motivated by analogous cardiac mapping work [46]. Given the relatively small sample size and the absence of LUTS questionnaires or functional urologic testing, these results are exploratory rather than intended to establish definitive age‐ and sex‐stratified reference ranges. No significant differences in 3D MRF T_1_ and T_2_ values were observed between men and women, consistent with T_1_ mapping work by Yalcin et al. [24] BWT tended to be higher in men, consistent with prior ultrasound studies [47, 48]. In this cohort, a positive association between T_2_ and subject age was observed at both 1.5 T and 3 T. Further research is needed to confirm these findings and clarify whether they reflect normal aging or occult age‐related LUTD. Moreover, larger studies are required to establish age‐ and sex‐stratified normative ranges for MRF bladder relaxation times.

### 4.4 Bladder Filling State

Subjects voided immediately before each MRI exam to maximize bladder wall thickness and reduce partial volume artifacts from urine. This protocol was designed for quantitative bladder wall mapping rather than focal lesion detection. Because the benign functional urologic conditions motivating this work often involve diffuse bladder wall remodeling, this emphasis was considered appropriate. Where lesion assessment is also required, future protocols could image at a defined filling state tailored to the clinical application. Even with the post‐void protocol, bladder filling state remained a potential confounder in this study. Variability in post‐void residual (PVR) and in time elapsed between voiding and image acquisition may contribute to measurement variability. Correia et al. reported increased T_1_ values with 2D MRF in the full versus empty bladder state but no change in T_2_ values [26]. However, given the lower‐resolution 2D acquisition, these differences may reflect partial volume averaging of the thin bladder wall during filling. Future work is needed to investigate the influence of filling state on bladder wall relaxation times. If such a dependency is observed, protocols could image at a consistent filling state or account for the PVR, which could be directly quantified from 3D whole‐bladder MRF maps when interpreting bladder wall relaxation times.

### 4.5 Synthetic Imaging

Synthetic imaging could streamline MRI exams by reducing the number of separate acquisitions, as images with adjustable contrast can be retrospectively derived from a single quantitative scan. In this study, synthetic T_2_‐weighted and T_1_‐weighted (PSIR) images were calculated from MRF maps and used to quantify BWT, with strong agreement relative to reference measurements from conventional scans. These contrasts were selected because T_2_‐weighted images are used clinically to assess bladder morphology. While PSIR images are not clinically acquired in the bladder [49], they can provide a FLAIR‐like contrast that nulls signal from urine to enhance bladder wall delineation. Qualitative differences were observed between synthetic and conventional T_2_‐weighted images. Notably, fat appeared hyperintense on conventional images due to J‐coupling effects in the FSE sequence, which were not simulated in the synthetic contrast [50]. Other discrepancies may reflect tissue properties, such as diffusion or magnetization transfer, that were not modeled in the MRF reconstruction [51].

### 4.6 Limitations and Future Directions

This study has several limitations. For this initial study, the acquisition was fully sampled along the partition direction to minimize aliasing artifacts, resulting in a relatively long scan time (11.8 min) that may increase susceptibility to bulk motion, respiration, bowel peristalsis, and passive bladder filling, particularly in the dome and posterior wall. Future work will aim to reduce scan time by shortening the MRF sequence and undersampling along the partition direction. Because partition undersampling would require a true 3D DIP reconstruction, it would no longer be compatible with the current slice‐by‐slice 2D DIP approach. Another limitation is the computational burden of DIP because network training is performed de novo. DIP requires approximately 2 h to reconstruct each slice, which remains a major barrier to clinical deployment. In this feasibility study, the added computation time was considered acceptable because DIP provided better map quality than conventional reconstructions. Future work will focus on reducing computation time, including initializing network weights from adjacent slices or leveraging recent work from our group using meta‐learning to accelerate DIP reconstructions for cardiac MRF [52].

Improving the spatial resolution, particularly along the partition direction, is an area of interest for future work to minimize partial volume artifacts. Additionally, off‐resonance deblurring was not performed, although the spiral readout was kept short (3.5 ms) to minimize blurring. The absence of fat suppression in the MRF scan is a potential source of error, as perivesical fat can introduce partial volume averaging or chemical shift artifacts, particularly at 3 T. This likely contributed to the bright rim of elevated T_1_ values observed at interfaces between the bladder wall and perivesical fat at 3 T; this artifact was not apparent at 1.5 T (Supporting Figure S13). Simulation results suggest that signal contamination from fat is a plausible explanation for this artifact at 3 T (Supporting Figure S14). Voxels affected by this artifact were avoided during ROI placement for bladder wall measurements. Future protocols could incorporate fat‐water separation using Dixon [53] or rosette MRF techniques [54], which could eliminate these artifacts and provide quantitative fat fraction maps that may be useful for evaluating fat infiltration of the bladder wall.

Finally, bladder function (uroflowmetry and UDS) and LUTS were not assessed in this cohort despite a broad range of ages (18–78 years). The incidence and prevalence of LUTD, including emergence of symptoms, are known to increase with age. Therefore, some participants—particularly older subjects—may have had unrecognized LUTD, which could have affected bladder wall relaxation times and contributed to the positive association between T_2_ and age. This may be especially relevant in the dome and posterior wall, where age‐related structural changes, including diverticula, can occur. Future work will incorporate standardized LUTS questionnaires and functional urologic testing to better distinguish normal aging effects from occult LUTD.

## 5 Conclusion

Simultaneous 3D T_1_, T_2_, and M_0_ mapping of the bladder wall is feasible using a stack‐of‐spirals MRF acquisition with a deep image prior reconstruction. Quantitative relaxation times from MRF maps and bladder wall thickness from synthetic weighted images were characterized in healthy adults at both 1.5 T and 3 T. These findings provide a baseline for future translational studies applying 3D MRF to characterize pathological bladder wall remodeling in patient populations.

## Author Contributions

**S.W.:** study design, data collection and analysis, manuscript preparation. **G.I.:** study design, clinical context, manuscript review and editing. **M.O.:** image segmentation and analysis. **W.S.:** study design, clinical context, manuscript review and editing. **A.C.:** study design, clinical context, manuscript review and editing. **J.H.:** study design, MRF sequence and reconstruction development, data collection and analysis, manuscript preparation. All authors approved the final manuscript.

## Funding

This work was supported by the NIH (NHLBI R01HL163030 and NIDDK K12DK111011); the Society for Urodynamics, Female Pelvic Medicine, & Urogenital Reconstruction; and Siemens Healthineers.

## Conflicts of Interest

Jesse Hamilton and Shane Wells receive research grant support from Siemens Healthineers.

## Supporting information


**Figure S1:** Overview of the deep image prior reconstruction. (A) MRF subspace images and coil sensitivity maps are jointly estimated using a loss function that minimizes the mean squared error (MSE) relative to undersampled k‐space data. (B) The subspace images are passed through a multilayer perceptron (MLP), which estimates T_1_, T_2_, and complex‐valued M_0_ maps.
**Table S1:** Acquisition parameters for the bladder MRI protocol. MOLLI and T2‐prepared GRE maps were acquired for comparison using vendor‐provided product sequences (MyoMaps, Siemens Healthineers), with a bSSFP readout at 1.5 T and a FLASH readout at 3 T to reduce off‐resonance artifacts. Zero‐filling interpolation was applied to all images to improve the apparent spatial resolution. Both acquired (true) and zero‐filled (effective) voxel sizes are reported. For 3D MRF, zero‐filling interpolation was applied along all three axes, whereas for conventional 2D sequences it was only applied in‐plane.
**Figure S2:** ROI placement in a representative subject. (A) Co‐registered 3D MRF T1 and T2 maps are shown from a sagittal slice through the bladder midline, where ROIs were drawn in the trigone, anterior, posterior, dome, and base of the bladder wall. Two additional ROIs were placed in lateral sagittal slices approximately (B) 20 mm left and (C) right of the midline, aligned with the trigone. ROIs were drawn using ITK‐SNAP. The T1 and T2 maps were inherently co‐registered since they were derived from a single MRF acquisition, and thus, the same ROIs were used for both maps.
**Figure S3:** Representative 3D MRF T1, T2, and M0 maps from a 41‐year‐old male subject acquired at (A) 1.5 T and (B) 3 T. Note the difference in scale bars for T1 maps at each field strength. Maps are displayed over the full 300 × 300 mm2 field‐of‐view. Maps cropped to provide a zoomed‐in view of the bladder are shown in the main text in Figure 2.
**Figure S4:** 3D MRF T1, T2, and M0 maps acquired from an additional subject (44‐year‐old female) at (A) 1.5 T and (B) 3 T.
**Figure S5:** 3D MRF T1, T2, and M0 maps acquired from a second additional subject (28‐year‐old male) at (A) 1.5 T and (B) 3 T.
**Figure S6:** 3D MRF T1, T2, and M0 maps acquired from a third additional subject (20‐year‐old female) at (A) 1.5 T and (B) 3 T.
**Figure S7:** Effect of zero‐filling interpolation on 3D bladder MRF maps. MRF maps from a representative subject at 1.5 T were reconstructed using DIP. As described in the main manuscript, data were acquired with a voxel size of 1 × 1 × 3 mm3, matrix size 304 × 304, field‐of‐view 300 × 300 mm2, and 30 partitions. The resulting DIP maps are displayed (A) without zero‐filling interpolation and (B) with zero‐filling interpolation to an effective resolution of 0.5 × 0.5 × 1.5 mm3. In each panel, a cropped region centered on the bladder is shown together with a magnified view of the area indicated by the red dashed square. Zero‐filling interpolation does not change the acquired voxel size, but it improves the appearance of the maps, producing a smoother, less pixelated depiction of the bladder wall. Global bladder wall relaxation times measured in the displayed slice (mean ± standard deviation) were similar with and without interpolation: T1 950 ± 109 ms and T2 43.5 ± 6.5 ms without interpolation, versus T1 953 ± 102 ms and T2 43.7 ± 6.2 ms with interpolation.
**Table S2:** Summary of regional bladder T1 measurements. Mean values, intersubject variability (SD and CV), and spatial heterogeneity (as a measure of precision) are presented for (A) MRF and (B) MOLLI T1 measurements at 1.5 T and 3 T in specific bladder wall regions.
**Table S3:** Summary of regional bladder T2 measurements. Mean values, intersubject variability (SD and CV), and spatial heterogeneity (as a measure of precision) are presented for (A) MRF and (B) T2‐prepared GRE measurements at 1.5 T and 3 T in specific bladder wall regions.
**Table S4:** Summary of regional bladder wall thickness measurements. Mean values and intersubject variability (SD and CV) are reported for BWT from (A) synthetic T2‐weighted and PSIR images derived from MRF maps and (B) conventional T2‐weighted images at 1.5 T and 3 T.
**Figure S8:** Intersubject measurement variability by bladder region. Intersubject variability is shown within each bladder wall region for (A) T1, (B) T2, and (C) BWT using MRF and comparison sequences at 1.5 T and 3 T. Variability is reported as coefficients of variation (CV, %) for T1 and T2 to facilitate field strength comparisons and as standard deviations (SD, mm) for BWT.
**Figure S9:** Spatial heterogeneity with 3D bladder MRF and 2D single‐parametric mapping at 1.5 T and 3 T. Spatial CV values for global bladder wall (A) T1 and (B) T2 over all subjects are shown, with significant differences indicated by asterisks (*p < 0.05, **p < 0.01).
**Figure S10:** Spatial heterogeneity in tissue property estimates within specific bladder wall regions. Spatial CV values for (A) T1 and (B) T2 are reported as percentages within different bladder wall regions for 3D MRF and 2D single‐parametric mapping at 1.5 T and 3 T. For each field strength and acquisition, significant differences between bladder wall regions are indicated by color‐coded asterisks (*p < 0.05, **p < 0.01, ***p < 0.001).
**Figure S11:** Comparison of MRF relaxation times and BWT in male and female subjects. Regional measurements of (A, B) T1, (C, D) T2, and (E, F) BWT acquired with MRF at 1.5 T and 3 T are presented for male and female subjects. For each bladder region, significant sex differences are indicated by an asterisk (*p < 0.05, **p < 0.01, ***p < 0.001).
**Figure S12:** Association between bladder MRF relaxation times and BWT with age. Regional (A) T1, (B) T2, and (C) BWT measurements using MRF at 1.5 T and 3 T are displayed as a function of age. The best‐fit regression line is reported for each case, along with the coefficient of determination (R2 value) and p value. Shaded areas indicate the 95% confidence interval for the regression.
**Figure S13:** Bright rim artifact on a 3 T MRF T1 map. Representative MRF T1 and T2 maps from the same subject are shown at (A) 1.5 T and (B) 3 T. On the 3 T T1 map, a bright rim of elevated values is visible at the interface between the bladder wall and perivesical fat (arrows). This artifact is not apparent on the 3 T T2 map or the 1.5 T T1 and T2 maps.
**Figure S14:** Effect of partial volume averaging from fat on MRF bladder wall relaxation time measurements. Simulation results show (A,B) the estimated apparent T1 and T2 at 1.5 T and (C,D) at 3 T. Dotted lines indicate the simulated reference T1 and T2 values for pure bladder wall (blue) and pure fat (green).

## Data Availability

The data that support the findings of this study are available from the corresponding author upon reasonable request.

## References

[1] K. S. Coyne , C. C. Sexton , C. L. Thompson , et al., “The prevalence of lower urinary tract symptoms (LUTS) in the USA, the UK and Sweden: results from the Epidemiology of LUTS (EpiLUTS) study,” BJU International 104, no. 3 (2009): 352–360, 10.1111/j.1464-410X.2009.08427.x. 19281467

[2] H. Kannan , L. Radican , R. S. Turpin , and S. C. Bolge , “Burden of Illness Associated With Lower Urinary Tract Symptoms Including Overactive Bladder/Urinary Incontinence,” Urology 74, no. 1 (2009): 34–38, 10.1016/j.urology.2008.12.077. 19428076

[3] D. Cella , A. R. Smith , J. W. Griffith , et al., “A New Brief Clinical Assessment of Lower Urinary Tract Symptoms for Women and Men: LURN SI‐10,” Journal of Urology 203, no. 1 (2020): 164–170, 10.1097/JU.0000000000000465. 31364922 PMC6904437

[4] M. D. Eckhardt , G. E. van Venrooij , and T. A. Boon , “Symptoms and Quality of Life Versus Age, Prostate Volume, and Urodynamic Parameters in 565 Strictly Selected Men With Lower Urinary Tract Symptoms Suggestive of Benign Prostatic Hyperplasia,” Urology 57, no. 4 (2001): 695–700, 10.1016/s0090-4295(00)01101-8. 11306383

[5] A. Gammie and M. J. Drake , “The Fundamentals of Uroflowmetry Practice, Based on International Continence Society Good Urodynamic Practices Recommendations,” Neurourology and Urodynamics 37, no. S6 (2018): S44–S49, 10.1002/nau.23777. 30614059

[6] T. L. Frenkl , R. Railkar , J. Palcza , et al., “Variability of Urodynamic Parameters in Patients With Overactive Bladder,” Neurourology and Urodynamics 30, no. 8 (2011): 1565–1569, 10.1002/nau.21081. 21674594

[7] J. Y. Yeung , M. A. Eschenbacher , and R. N. Pauls , “Pain and Embarrassment Associated with Urodynamic Testing in Women,” International Urogynecology Journal 25, no. 5 (2014): 645–650, 10.1007/s00192-013-2261-1. 24280994

[8] V. Ficarra , M. Rossanese , M. Zazzara , et al., “The Role of Inflammation in Lower Urinary Tract Symptoms (LUTS) due to Benign Prostatic Hyperplasia (BPH) and Its Potential Impact on Medical Therapy,” Current Urology Reports 15, no. 12 (2014): 463, 10.1007/s11934-014-0463-9. 25312251

[9] C. H. Fry , D. G. Kitney , J. Paniker , M. J. Drake , A. Kanai , and K. E. Andersson , “Fibrosis and the Bladder, Implications for Function ICI‐RS 2017,” Neurourology and Urodynamics 37, no. S4 (2018): S7–S12, 10.1002/nau.23725. 30133788

[10] F. Fusco , M. Creta , C. De Nunzio , et al., “Progressive Bladder Remodeling due to Bladder Outlet Obstruction: A Systematic Review of Morphological and Molecular Evidences in Humans,” BMC Urology 18 (2018): 15, 10.1186/s12894-018-0329-4. 29519236 PMC5844070

[11] C. H. S. Bellucci , W. d. O. Ribeiro , T. S. Hemerly , et al., “Increased Detrusor Collagen Is Associated with Detrusor Overactivity and Decreased Bladder Compliance in Men with Benign Prostatic Obstruction,” Prostate International 5, no. 2 (2017): 70–74, 10.1016/j.prnil.2017.01.008. 28593170 PMC5448720

[12] X. Yang , Q. Pu , Y. Wen , et al., “Activation of the TGF‐β1/Smads/α‐SMA Pathway Is Related to Histological and Functional Changes in Children With Neurogenic Bladder,” Scientific Reports 12, no. 1 (2022): 9285, 10.1038/s41598-022-13470-0. 35662268 PMC9166803

[13] E. Compérat , A. Reitz , A. Delcourt , F. Capron , P. Denys , and E. Chartier‐Kastler , “Histologic Features in the Urinary Bladder Wall Affected from Neurogenic Overactivity—A Comparison of Inflammation, Oedema and Fibrosis With and Without Injection of Botulinum Toxin Type A,” European Urology 50, no. 5 (2006): 1058–1064, 10.1016/j.eururo.2006.01.025. 16517054

[14] C. Jia , T. Xing , Z. Shang , X. Cui , Q. Wang , and T. Ou , “Botulinum Toxin A Improves Neurogenic Bladder Fibrosis by Suppressing Transforming Growth Factor β1 Expression in Rats,” Translational Andrology and Urology 10, no. 5 (2021): 2000–2007, 10.21037/tau-21-62. 34159080 PMC8185670

[15] S. Bull , S. K. White , S. K. Piechnik , et al., “Human Non‐Contrast T1 Values and Correlation With Histology in Diffuse Fibrosis,” Heart 99, no. 13 (2013): 932–937, 10.1136/heartjnl-2012-303052. 23349348 PMC3686317

[16] E. Dall'Armellina , S. K. Piechnik , V. M. Ferreira , et al., “Cardiovascular Magnetic Resonance by Non Contrast T1‐Mapping Allows Assessment of Severity of Injury in Acute Myocardial Infarction,” Journal of Cardiovascular Magnetic Resonance: Official Journal of the Society for Cardiovascular Magnetic Resonance 14, no. 1 (2012): 15, 10.1186/1532-429X-14-15. 22309452 PMC3312869

[17] R. Hinojar , E. Nagel , and V. O. Puntmann , “T1 Mapping in Myocarditis ‐ Headway to a New Era for Cardiovascular Magnetic Resonance,” Expert Review of Cardiovascular Therapy 13, no. 8 (2015): 871–874, 10.1586/14779072.2015.1051035. 26018769

[18] T. D. Karamitsos , S. K. Piechnik , S. M. Banypersad , et al., “Noncontrast T1 Mapping for the Diagnosis of Cardiac Amyloidosis,” JACC: Cardiovascular Imaging 6, no. 4 (2013): 488–497, 10.1016/j.jcmg.2012.11.013. 23498672

[19] S. Pica , D. M. Sado , V. Maestrini , et al., “Reproducibility of Native Myocardial T1 Mapping in the Assessment of Fabry Disease and Its Role in Early Detection of Cardiac Involvement by Cardiovascular Magnetic Resonance,” Journal of Cardiovascular Magnetic Resonance: Official Journal of the Society for Cardiovascular Magnetic Resonance 16, no. 1 (2014): 99, 10.1186/s12968-014-0099-4. 25475749 PMC4256727

[20] S. Giri , Y. C. Chung , A. Merchant , et al., “T2 Quantification for Improved Detection of Myocardial Edema,” Journal of Cardiovascular Magnetic Resonance 11, no. 1 (2009): 56, 10.1186/1532-429X-11-56. 20042111 PMC2809052

[21] C. H. Park , E. Y. Choi , H. M. Kwon , et al., “Quantitative T2 Mapping for Detecting Myocardial Edema After Reperfusion of Myocardial Infarction: Validation and Comparison With T2‐Weighted Images,” International Journal of Cardiovascular Imaging 29, no. Suppl 1 (2013): 65–72, 10.1007/s10554-013-0256-0. 23765068

[22] P. Tyagi , J. Janicki , C. H. Moon , J. Kaufman , and C. Chermansky , “Novel Contrast Mixture Achieves Contrast Resolution of Human Bladder Wall Suitable for T1 Mapping: Applications in Interstitial Cystitis and Beyond,” International Urology and Nephrology 50, no. 3 (2018): 401–409, 10.1007/s11255-018-1794-0. 29392488 PMC6028942

[23] L. Ye , Y. Wang , W. Xiang , J. Yao , J. Liu , and B. Song , “Radiomic Analysis of Quantitative T2 Mapping and Conventional MRI in Predicting Histologic Grade of Bladder Cancer,” Journal of Clinical Medicine 12, no. 18 (2023): 5900, 10.3390/jcm12185900. 37762841 PMC10531568

[24] A. Yalcin , M. H. Gultekin , A. Erdogan , and B. Y. Cankaya , “Signal Abnormalities of the Bladder Wall Detected by Native T(1) Mapping in Patients With Overactive Bladder,” NMR in Biomedicine 35, no. 9 (2022): e4748, 10.1002/nbm.4748. 35466455

[25] D. Ma , V. Gulani , N. Seiberlich , et al., “Magnetic Resonance Fingerprinting,” Nature 495, no. 7440 (2013): 187–192, 10.1038/nature11971. 23486058 PMC3602925

[26] E. T. d. O. Correia , J. Badreddine , R. Boyacioglu , et al., “Quantitative Assessment of Bladder Tissue Properties Using Magnetic Resonance Fingerprinting: A Pilot Feasibility Study in Healthy Volunteers,” Radiologia Brasileira 58 (2025): e20240104, 10.1590/0100-3984.2024.0104. 40391153 PMC12087349

[27] J. I. Hamilton , Y. Jiang , Y. Chen , et al., “MR Fingerprinting for Rapid Quantification of Myocardial T1, T2, and Proton Spin Density,” Magnetic Resonance in Medicine 77, no. 4 (2017): 1446–1458, 10.1002/mrm.26216. 27038043 PMC5045735

[28] J. H. Brittain , B. S. Hu , G. A. Wright , C. H. Meyer , A. Macovski , and D. G. Nishimura , “Coronary Angiography With Magnetization‐Prepared T2 Contrast,” Magnetic Resonance in Medicine 33, no. 5 (1995): 689–696, 10.1002/mrm.1910330515. 7596274

[29] B. Hargreaves , Variable‐Density Spiral Design Functions (Stanford University Magnetic Resonance Systems Research Laboratory, 2005), http://mrsrl.stanford.edu/~brian/vdspiral/.

[30] J. I. Hamilton , Y. Jiang , D. Ma , et al., “Investigating and Reducing the Effects of Confounding Factors for Robust T1 and T2 Mapping With Cardiac MR Fingerprinting,” Magnetic Resonance Imaging 53 (2018): 40–51, 10.1016/j.mri.2018.06.018. 29964183 PMC7755105

[31] D. F. McGivney , E. Pierre , D. Ma , et al., “SVD Compression for Magnetic Resonance Fingerprinting in the Time Domain,” IEEE Transactions on Medical Imaging 33, no. 12 (2014): 2311–2322, 10.1109/TMI.2014.2337321. 25029380 PMC4753055

[32] D. Kim , S. F. Cauley , K. S. Nayak , R. M. Leahy , and J. P. Haldar , “Region‐Optimized Virtual (ROVir) Coils: Localization and/or Suppression of Spatial Regions Using Sensor‐Domain Beamforming,” Magnetic Resonance in Medicine 86, no. 1 (2021): 197–212, 10.1002/mrm.28706. 33594732 PMC8248187

[33] D. Ulyanov , A. Vedaldi , and V. Lempitsky , “Deep Image Prior,” in Proceedings of the IEEE Computer Society Conference on Computer Vision and Pattern Recognition (IEEE Computer Society, 2018), 9446–9454.

[34] J. I. Hamilton , “A Self‐Supervised Deep Learning Reconstruction for Shortening the Breathhold and Acquisition Window in Cardiac Magnetic Resonance Fingerprinting,” Frontiers in Cardiovascular Medicine 9 (2022): 928546, 10.3389/fcvm.2022.928546. 35811730 PMC9260051

[35] J. Fessler and B. Sutton , “Nonuniform Fast Fourier Transforms Using Min‐Max Interpolation,” IEEE Transactions on Signal Processing 51, no. 2 (2003): 560–574.

[36] G. Lima da Cruz , A. Bustin , O. Jaubert , T. Schneider , R. M. Botnar , and C. Prieto , “Sparsity and Locally Low Rank Regularization for MR Fingerprinting,” Magnetic Resonance in Medicine 81, no. 6 (2019): 3530–3543, 10.1002/mrm.27665. 30720209 PMC6492150

[37] D. R. Messroghli , J. C. Moon , V. M. Ferreira , et al., “Clinical Recommendations for Cardiovascular Magnetic Resonance Mapping of T1, T2, T2* and Extracellular Volume: A Consensus Statement by the Society for Cardiovascular Magnetic Resonance (SCMR) Endorsed by the European Association for Cardiovascular Imaging,” Journal of Cardiovascular Magnetic Resonance 19, no. 1 (2017): 75. 28992817 10.1186/s12968-017-0389-8 PMC5633041

[38] P. A. Yushkevich , J. Piven , H. C. Hazlett , et al., “User‐Guided 3D Active Contour Segmentation of Anatomical Structures: Significantly Improved Efficiency and Reliability,” NeuroImage 31, no. 3 (2006): 1116–1128, 10.1016/j.neuroimage.2006.01.015. 16545965

[39] J. M. Bland and D. G. Altman , “Statistical Methods for Assessing Agreement Between Two Methods of Clinical Measurement,” Lancet (London, England) 1, no. 8476 (1986): 307–310. 2868172

[40] T. Hilbert , D. Xia , K. T. Block , et al., “Magnetization Transfer in Magnetic Resonance Fingerprinting,” Magnetic Resonance in Medicine 84, no. 1 (2020): 128–141, 10.1002/mrm.28096. 31762101 PMC7083689

[41] J. Assländer , S. J. Glaser , and J. Hennig , “Pseudo Steady‐State Free Precession for MR‐Fingerprinting,” Magnetic Resonance in Medicine 77, no. 3 (2017): 1151–1161, 10.1002/mrm.26202. 27079826

[42] P. Kellman and M. S. Hansen , “T1‐Mapping in the Heart: Accuracy and Precision,” Journal of Cardiovascular Magnetic Resonance 16, no. 1 (2014): 2, 10.1186/1532-429X-16-2. 24387626 PMC3927683

[43] M. D. Cerqueira , “Standardized Myocardial Segmentation and Nomenclature for Tomographic Imaging of the Heart: A Statement for Healthcare Professionals From the Cardiac Imaging Committee of the Council on Clinical Cardiology of the American Heart Association,” Circulation 105, no. 4 (2002): 539–542, 10.1161/hc0402.102975. 11815441

[44] L. E. Anzia , C. J. Johnson , L. Mao , et al., “Comprehensive Non‐Invasive Analysis of Lower Urinary Tract Anatomy Using MRI,” Abdominal Radiology 46, no. 4 (2021): 1670–1676, 10.1007/s00261-020-02808-9. 33040167 PMC8036233

[45] L. Shahid , J. P. Gonzalez‐Pereira , C. Johnson , W. Bushman , and A. Roldán‐Alzate , “Computational Fluid Dynamics of Bladder Voiding Using 3D Dynamic MRI,” International Journal for Numerical Methods in Biomedical Engineering 40, no. 9 (2024): e3850, 10.1002/cnm.3850. 39010679 PMC12684817

[46] C. Roy , A. Slimani , C. de Meester , et al., “Age and Sex Corrected Normal Reference Values of T1, T2 T2* and ECV in Healthy Subjects at 3T CMR,” Journal of Cardiovascular Magnetic Resonance 19, no. 1 (2017): 72, 10.1186/s12968-017-0371-5. 28934962 PMC5609021

[47] O. W. Hakenberg , C. Linne , A. Manseck , and M. P. Wirth , “Bladder Wall Thickness in Normal Adults and Men With Mild Lower Urinary Tract Symptoms and Benign Prostatic Enlargement,” Neurourology and Urodynamics 19, no. 5 (2000): 585–593, 10.1002/1520-6777(2000)19:5<585::aid-nau5>3.0.co;2-u. 11002301

[48] A. I. Volikova , B. J. Marshall , J. M. A. Yin , R. Goodwin , P. E. P. Chow , and M. J. Wise , “Structural, Biomechanical and Hemodynamic Assessment of the Bladder Wall in Healthy Subjects,” Research and Reports in Urology 11 (2019): 233–245, 10.2147/RRU.S205383. 31565652 PMC6732741

[49] B. L. Daniel , A. Shimakawa , M. R. Blum , and R. J. Herfkens , “Single‐Shot Fluid Attenuated Inversion Recovery (FLAIR) Magnetic Resonance Imaging of the Bladder,” Journal of Magnetic Resonance Imaging 11, no. 6 (2000): 673–677, 10.1002/1522-2586(200006)11:6<673::AID-JMRI14>3.0.CO;2-F. 10862067

[50] R. M. Henkelman , P. A. Hardy , J. E. Bishop , C. S. Poon , and D. B. Plewes , “Why Fat Is Bright in RARE and Fast Spin‐Echo Imaging,” Journal of Magnetic Resonance Imaging 2, no. 5 (1992): 533–540, 10.1002/jmri.1880020511. 1392246

[51] O. Nykänen , M. Nevalainen , V. Casula , et al., “Deep‐Learning‐Based Contrast Synthesis From MRF Parameter Maps in the Knee Joint,” Journal of Magnetic Resonance Imaging 58, no. 2 (2023): 559–568, 10.1002/jmri.28573. 36562500 PMC10287835

[52] Z. Liu , C. Sheagren , Z. Liu , N. Seiberlich , L. Shen , and J. Hamilton , An Accelerated Deep Image Prior Reconstruction for Cardiac MR Fingerprinting Using Meta‐Learning (ISMRM, 2026), http://echo.ismrm.org/abstracts/view/855cf411‐f8ea‐4fcb‐9724‐5e91828c5798.

[53] O. Jaubert , G. Cruz , A. Bustin , et al., “Water–Fat Dixon Cardiac Magnetic Resonance Fingerprinting,” Magnetic Resonance in Medicine 83, no. 6 (2019): 2107–2123, 10.1002/mrm.28070. 31736146 PMC7064906

[54] Y. Liu , J. Hamilton , Y. Jiang , and N. Seiberlich , “Cardiac MRF Using Rosette Trajectories for Simultaneous Myocardial T1, T2, and Proton Density Fat Fraction Mapping,” Frontiers in Cardiovascular Medicine 9 (2022): 9, 10.3389/fcvm.2022.977603. PMC9530568 36204572

