# Supplementary material for: 3D MR Fingerprinting for Quantitative Bladder Wall T1, T2, and M0 Mapping in Healthy Subjects at 1.5 T and 3 T
Source: NMR Biomed. 2026 Jul 28;39(9):e70366. doi: 10.1002/nbm.70366 (PMC13411193; doi:10.1002/nbm.70366)
Supplement: Supplementary file 1 — Figure S1: Overview of the deep image prior reconstruction. (A) MRF subspace images and coil sensitivity maps are jointly estimated using a loss function that minimizes the mean squared error (MSE) relative to undersampled k‐space data. (B) The subspace images are passed through a multilayer perceptron (MLP), which estimates T1, T2, and complex‐valued M0 maps. Table S1: Acquisition parameters for the bladder MRI protocol. MOLLI and T2‐prepared GRE maps were acquired for comparison using vendor‐provided product sequences (MyoMaps, Siemens Healthineers), with a bSSFP readout at 1.5 T and a FLASH readout at 3 T to reduce off‐resonance artifacts. Zero‐filling interpolation was applied to all images to improve the apparent spatial resolution. Both acquired (true) and zero‐filled (effective) voxel sizes are reported. For 3D MRF, zero‐filling interpolation was applied along all three axes, whereas for conventional 2D sequences it was only applied in‐plane. Figure S2: ROI placement in a representative subject. (A) Co‐registered 3D MRF T1 and T2 maps are shown from a sagittal slice through the bladder midline, where ROIs were drawn in the trigone, anterior, posterior, dome, and base of the bladder wall. Two additional ROIs were placed in lateral sagittal slices approximately (B) 20 mm left and (C) right of the midline, aligned with the trigone. ROIs were drawn using ITK‐SNAP. The T1 and T2 maps were inherently co‐registered since they were derived from a single MRF acquisition, and thus, the same ROIs were used for both maps. Figure S3: Representative 3D MRF T1, T2, and M0 maps from a 41‐year‐old male subject acquired at (A) 1.5 T and (B) 3 T. Note the difference in scale bars for T1 maps at each field strength. Maps are displayed over the full 300 × 300 mm2 field‐of‐view. Maps cropped to provide a zoomed‐in view of the bladder are shown in the main text in Figure 2. Figure S4: 3D MRF T1, T2, and M0 maps acquired from an additional subject (44‐year‐old female) at (A) 1.5 T a [file NBM-39-e70366-s001.docx]

Supporting Information

# 1. Deep Image Prior Reconstruction

An overview of the Deep Image Prior (DIP) MRF reconstruction is provided here. Additional details can be found in *Hamilton JI. A Self-Supervised Deep Learning Reconstruction for Shortening the Breathhold and Acquisition Window in Cardiac Magnetic Resonance Fingerprinting. Front Cardiovasc Med 2022; 9:928546*. This approach combines a low-rank MRF subspace approximation with zero-shot (instance-specific) deep learning to reconstruct tissue property maps without external training data. A schematic of the reconstruction is shown in **Supporting Figure S1**. A fixed tensor $z\in\mathbb{R}^{N_{y}\times N_{x}\times d}$ containing uniform random numbers between 0-1 is input to a randomly initialized U-Net, where $N_{y}$ and $N_{x}$ are the image dimensions and $d$ controls the number of input feature channels (set here to $d=32$). The network output has size $N_{y}\times N_{x}\times2K$, where $K$ is the rank of the compressed MRF dictionary, and the factor 2 accounts for the interleaved real and imaginary parts. This quantity is reshaped to produce the complex-valued MRF subspace images, $x_{K}\in\mathbb{C}^{N_{y}\times N_{x}\times K}$.

The DIP reconstruction also jointly estimates coil sensitivity maps using a convolutional neural network (CNN). This CNN takes an initial estimate of the sensitivity maps as input, obtained by applying ESPIRiT to the time-averaged k-space data, and outputs refined sensitivity maps $S\in\mathbb{C}^{N_{y}\times N_{x}\times N_{c}}$, where $N_{c}$ is the number of coils. The subspace images and sensitivity maps are multiplied together, and the resulting images are multiplied by the conjugate transpose of the right singular matrix from the SVD of the dictionary ($V_{K}^{*}$) to yield uncompressed time-series images. Spiral k-space sampling is then performed using the non-uniform Fast Fourier Transform (NUFFT). The image reconstruction U-Net and sensitivity map CNN are jointly trained using a loss function that minimizes the mean squared error (MSE) between predicted and acquired k-space data, weighted by the spiral density compensation function (DCF). To avoid memory overflow, the loss is evaluated over mini-batches of $T$ time frames rather than all TRs.

Concurrently, a multilayer perceptron (MLP) is trained to estimate tissue property maps from the subspace images. The subspace images are vectorized to size $N_{y}N_{x}\times2K$, with voxels treated as the batch dimension, and passed through two fully connected layers with 300 nodes each, producing maps of T_1_, T_2_, and the real and imaginary parts of the proton density (M_0_). This MLP is trained in a self-supervised manner as follows: (1) the T_1_ and T_2_ maps are passed through a “fingerprint generator network”—another MLP with frozen weights pre-trained using the MRF dictionary to produce SVD-compressed fingerprints for arbitrary T_1_ and T_2_ combinations; (2) the simulated fingerprint at each voxel is multiplied by the complex M_0_ scaling factor, yielding synthetic subspace images; and (3) the MSE loss is computed relative to the subspace images generated by the U-Net in the step above to update the tissue property estimation MLP.

The DIP reconstruction was implemented in TensorFlow with a Keras backend. To mitigate overfitting to noise, 5% dropout was applied after each convolutional layer of the U-Net. Training was performed using an Adam optimizer (learning rate 0.001) for 300 epochs with a mini-batch size of 16 TRs, where one epoch corresponded to a complete pass over all 1460 TRs (91 iterations per epoch). As described in the main text, a 1D FFT was first applied along the partition direction of the 3D MRF k-space data, and each slice was then reconstructed independently using this 2D DIP framework.


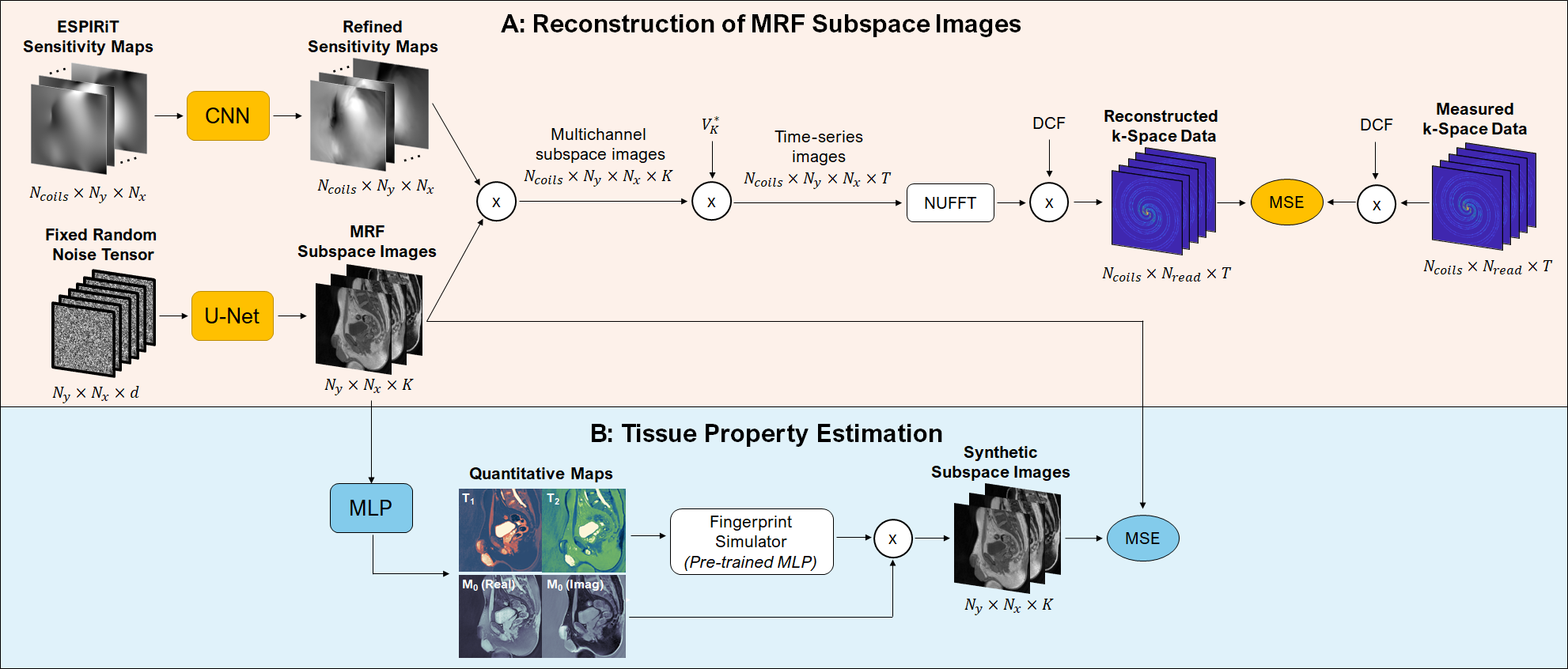


**Supporting Figure S1:** ***Overview of the Deep Image Prior reconstruction.* (A)** MRF subspace images and coil sensitivity maps are jointly estimated using a loss function that minimizes the mean squared error (MSE) relative to undersampled k-space data. **(B)** The subspace images are passed through a multilayer perceptron (MLP), which estimates T_1_, T_2_, and complex-valued M_0_ maps.

# 2. MRI Acquisition Parameters


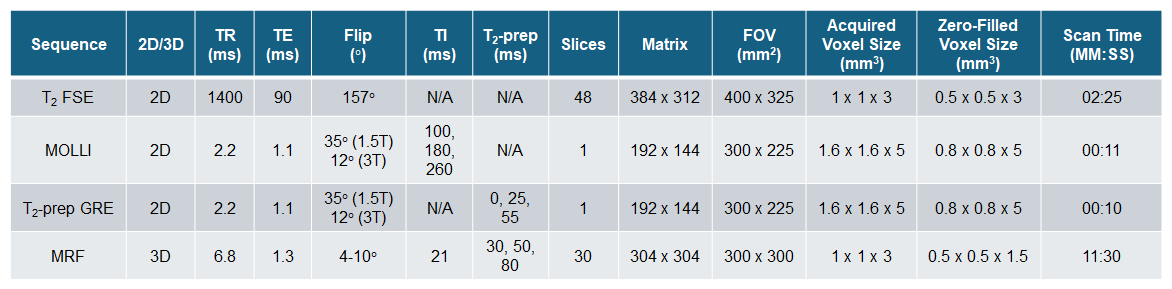


**Supporting Table S1:** ***Acquisition parameters for the bladder MRI protocol.*** MOLLI and T_2_-prepared GRE maps were acquired for comparison using vendor-provided product sequences (MyoMaps, Siemens Healthineers), with a bSSFP readout at 1.5T and a FLASH readout at 3T to reduce off-resonance artifacts. Zero-filling interpolation was applied to all images to improve the apparent spatial resolution. Both acquired (true) and zero-filled (effective) voxel sizes are reported. For 3D MRF, zero-filling interpolation was applied along all three axes, whereas for conventional 2D sequences it was only applied in-plane.

# 3. Segmentation of the Bladder Wall

**Supporting Figure S2: *ROI placement in a representative subject.*** **(A)** Co-registered 3D MRF T_1_ and T_2_ maps are shown from a sagittal slice through the bladder midline, where ROIs were drawn in the trigone, anterior, posterior, dome, and base of the bladder wall. Two additional ROIs were placed in lateral sagittal slices approximately **(B)** 20 mm left and **(C)** right of the midline, aligned with the trigone. ROIs were drawn using ITK-SNAP. The T_1_ and T_2_ maps were inherently co-registered since they were derived from a single MRF acquisition, and thus the same ROIs were used for both maps.

# 4. 3D MRF Maps (Full FOV)


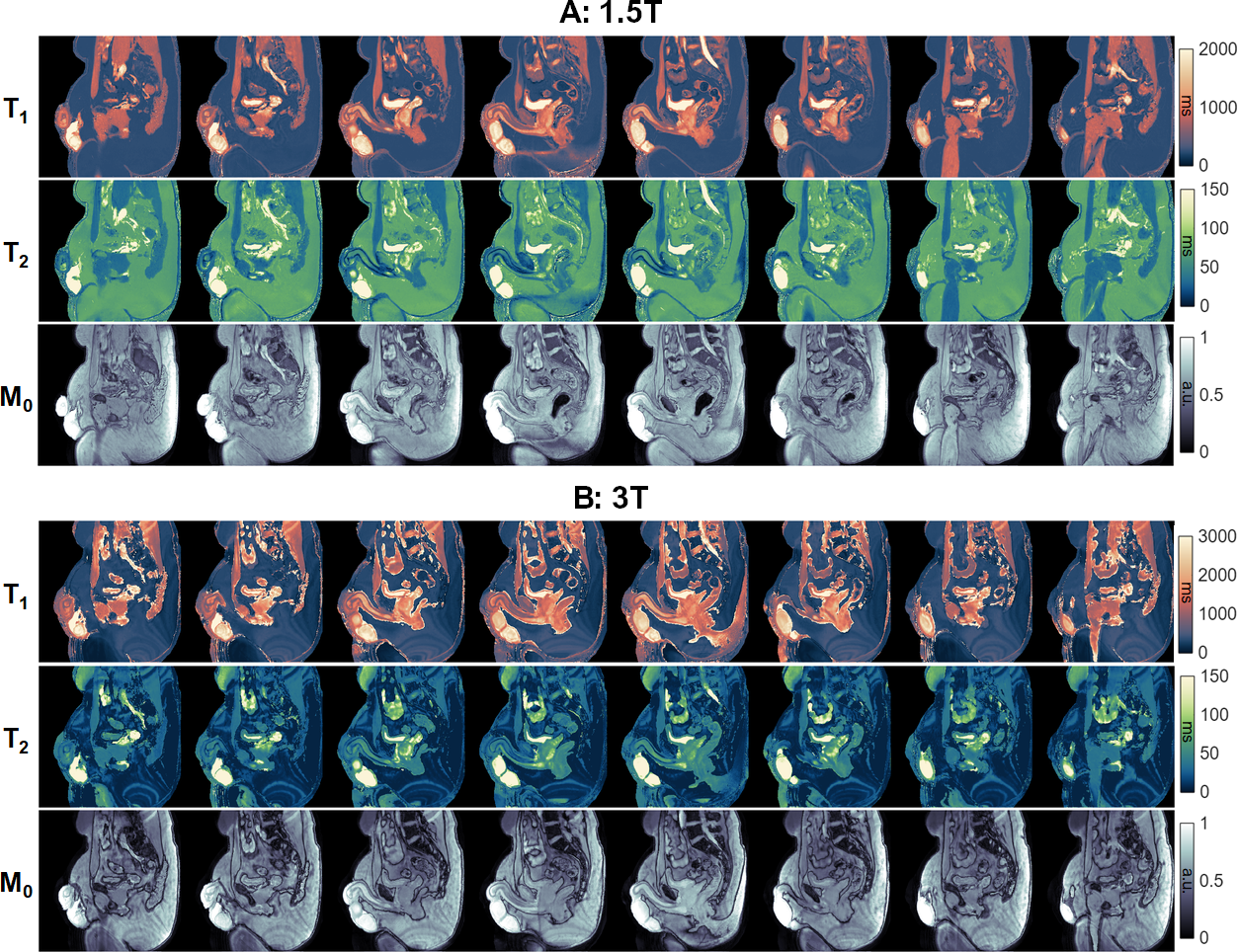


**Supporting Figure S3:** ***Representative 3D MRF T_1_, T_2_, and M_0_ maps from a 41-year-old male subject acquired at (A) 1.5T and (B) 3T.*** Note the difference in scale bars for T_1_ maps at each field strength. Maps are displayed over the full 300 x 300 mm^2^ field-of-view. Maps cropped to provide a zoomed-in view of the bladder are shown in the main text in **Fig. 2**.

# 5. 3D MRF Maps in Additional Subjects


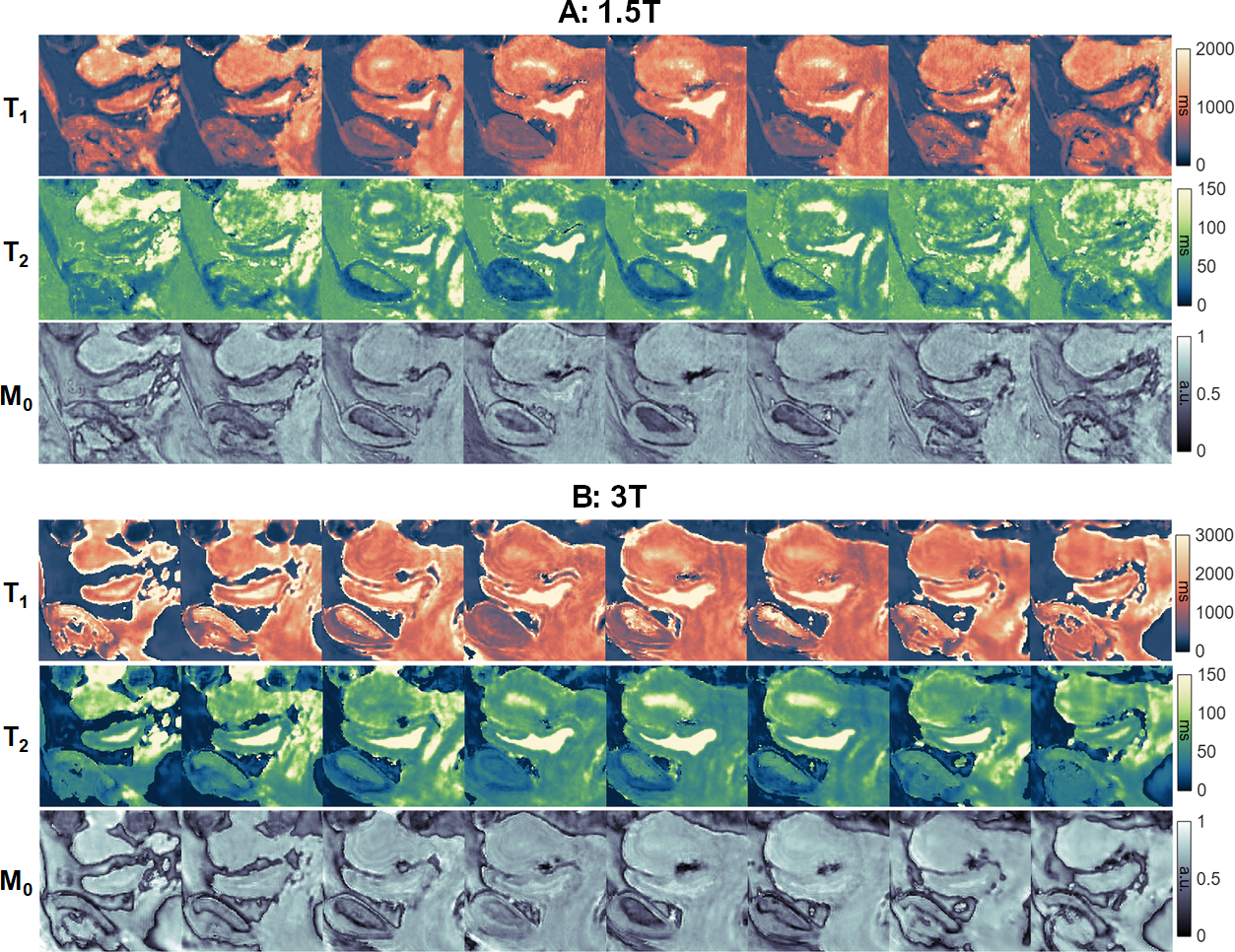


**Supporting Figure S4:** ***3D MRF T_1_, T_2_, and M_0_ maps acquired from an additional subject (44-year-old female) at (A) 1.5T and (B) 3T.***


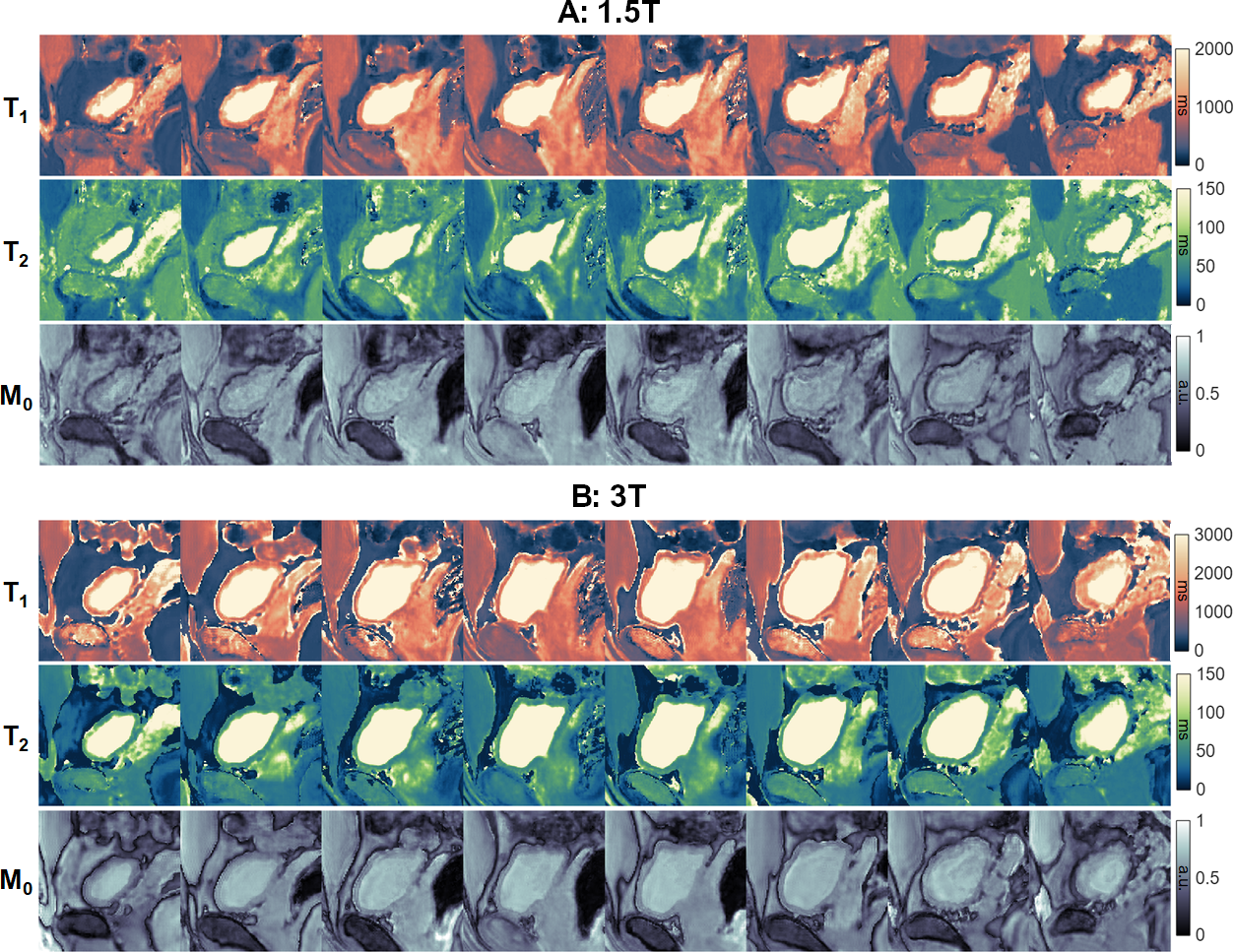


**Supporting Figure S5:** ***3D MRF T_1_, T_2_, and M_0_ maps acquired from a second additional subject (28-year-old male) at (A) 1.5T and (B) 3T.***

**Supporting Figure S6:** ***3D MRF T_1_, T_2_, and M_0_ maps acquired from a third additional subject (20-year-old female) at (A) 1.5T and (B) 3T.***

# 6. Effect of Zero-Filling Interpolation on Bladder MRF Maps

**Supporting Figure S7: *Effect of zero-filling interpolation on 3D bladder MRF maps***. MRF maps from a representative subject at 1.5T were reconstructed using DIP. As described in the main manuscript, data were acquired with a voxel size of 1 x 1 x 3 mm^3^, matrix size 304 x 304, field-of-view 300 x 300 mm^2^, and 30 partitions. The resulting DIP maps are displayed **(A)** without zero-filling interpolation and **(B)** with zero-filling interpolation to an effective resolution of 0.5 x 0.5 x 1.5 mm^3^. In each panel, a cropped region centered on the bladder is shown together with a magnified view of the area indicated by the red dashed square. Zero-filling interpolation does not change the acquired voxel size, but it improves the appearance of the maps, producing a smoother, less pixelated depiction of the bladder wall. Global bladder wall relaxation times measured in the displayed slice (mean $\pm$ standard deviation) were similar with and without interpolation: T_1_ 950 $\pm$ 109 ms and T_2_ 43.5 $\pm$ 6.5 ms without interpolation, versus T_1_ 953 $\pm$ 102 ms and T_2_ 43.7 $\pm$ 6.2 ms with interpolation.

# 7. Summary of Regional T_1_, T_2_, and BWT Measurements

**Supporting Table S2**: ***Summary of regional bladder T_1_ measurements.*** Mean values, intersubject variability (SD and CV), and spatial heterogeneity (as a measure of precision) are presented for **(A)** MRF and **(B)** MOLLI T_1_ measurements at 1.5T and 3T in specific bladder wall regions.

**Supporting Table S3**: ***Summary of regional bladder T_2_ measurements.*** Mean values, intersubject variability (SD and CV), and spatial heterogeneity (as a measure of precision) are presented for **(A)** MRF and **(B)** T_2_-prepared GRE measurements at 1.5T and 3T in specific bladder wall regions.

**Supporting Table S4**: ***Summary of regional bladder wall thickness measurements.*** Mean values and intersubject variability (SD and CV) are reported for BWT from **(A)** synthetic T_2_-weighted and PSIR images derived from MRF maps and **(B)** conventional T_2_-weighted images at 1.5T and 3T.

# 8. Intersubject Variability by Bladder Wall Region


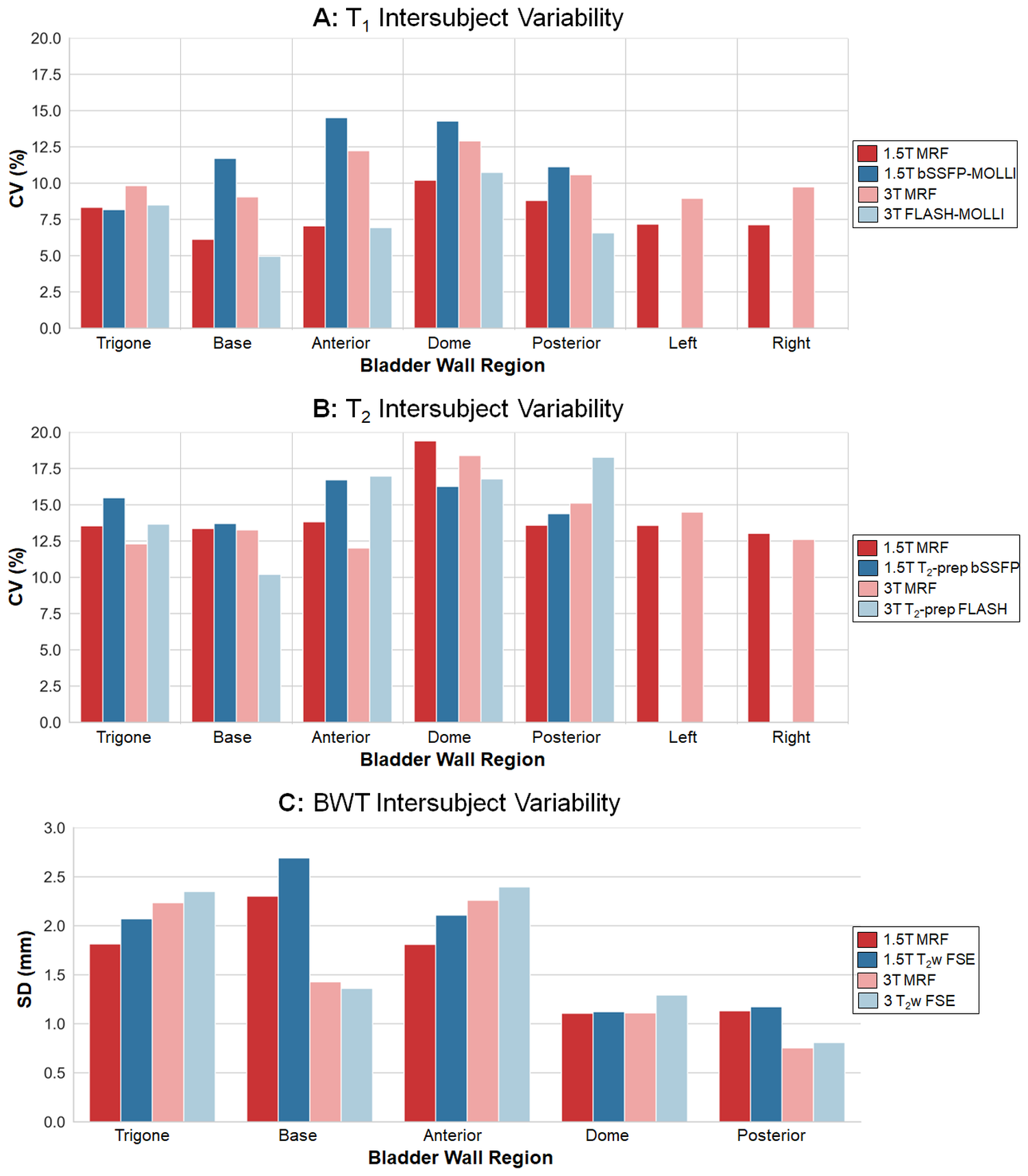


**Supporting Figure S8:** ***Intersubject measurement variability by bladder region.*** Intersubject variability is shown within each bladder wall region for **(A)** T_1_, **(B)** T_2_, and **(C)** BWT using MRF and comparison sequences at 1.5T and 3T. Variability is reported as coefficients of variation (CV, %) for T_1_ and T_2_ to facilitate field strength comparisons and as standard deviations (SD, mm) for BWT.

# 9. Spatial Heterogeneity (Intrasubject Variability)

**Supporting Figure S9:** **Spatial heterogeneity with 3D bladder MRF and 2D single-parametric mapping at 1.5T and 3T.** Spatial CV values for global bladder wall (A) T_1_ and (B) T_2_ over all subjects are shown, with significant differences indicated by asterisks (* p < 0.05, ** p < 0.01).

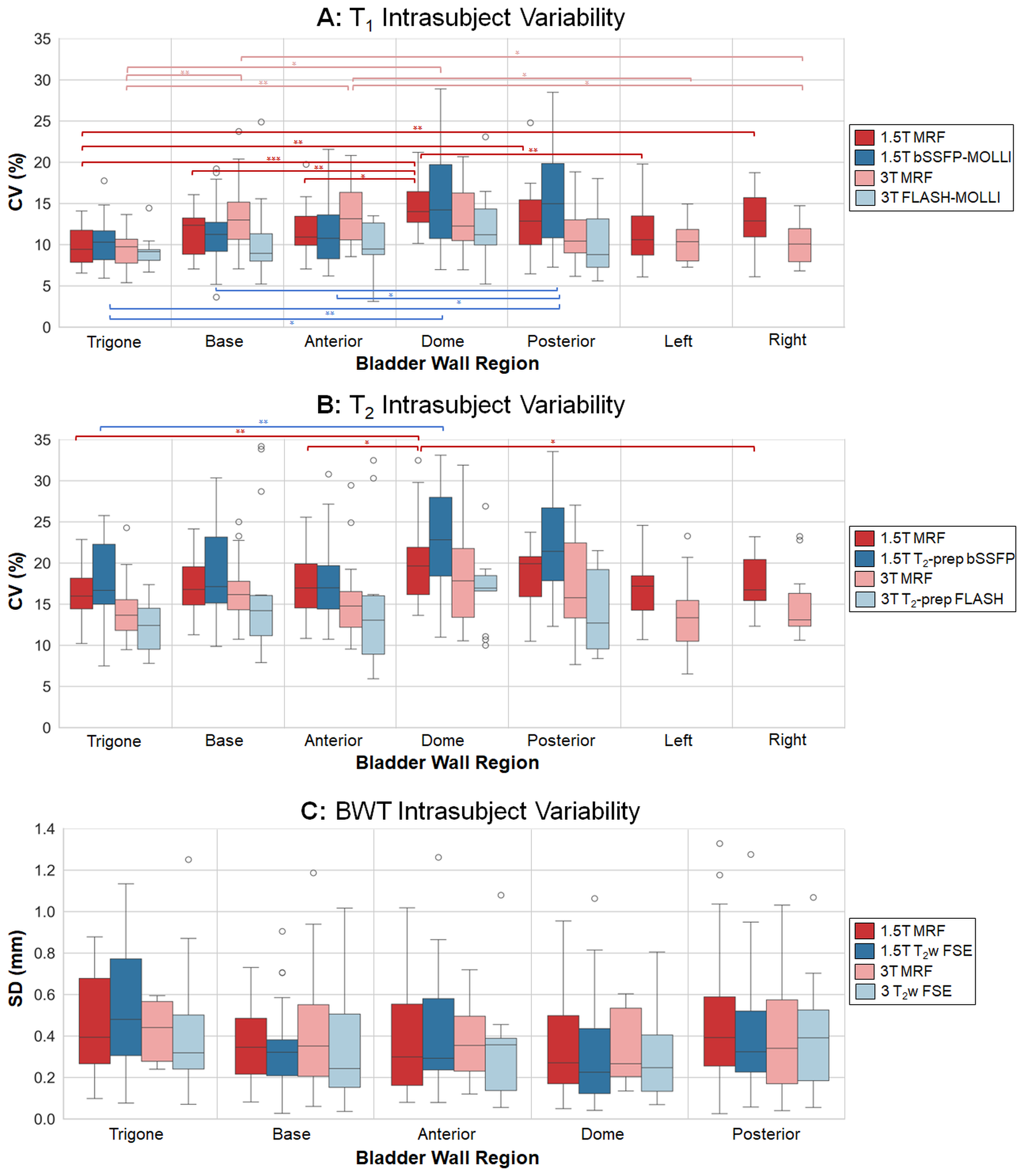


**Supporting Figure S10:** ***Spatial heterogeneity in tissue property estimates within specific bladder wall regions.*** Spatial CV values for **(A)** T_1_ and **(B)** T_2_ are reported as percentages within different bladder wall regions for 3D MRF and 2D single-parametric mapping at 1.5T and 3T. For each field strength and acquisition, significant differences between bladder wall regions are indicated by color-coded asterisks (* *p* < 0.05, ** *p* < 0.01, *** *p* < 0.001).

# 10. Sex Differences in Regional Bladder T_1_, T_2_, and BWT Values


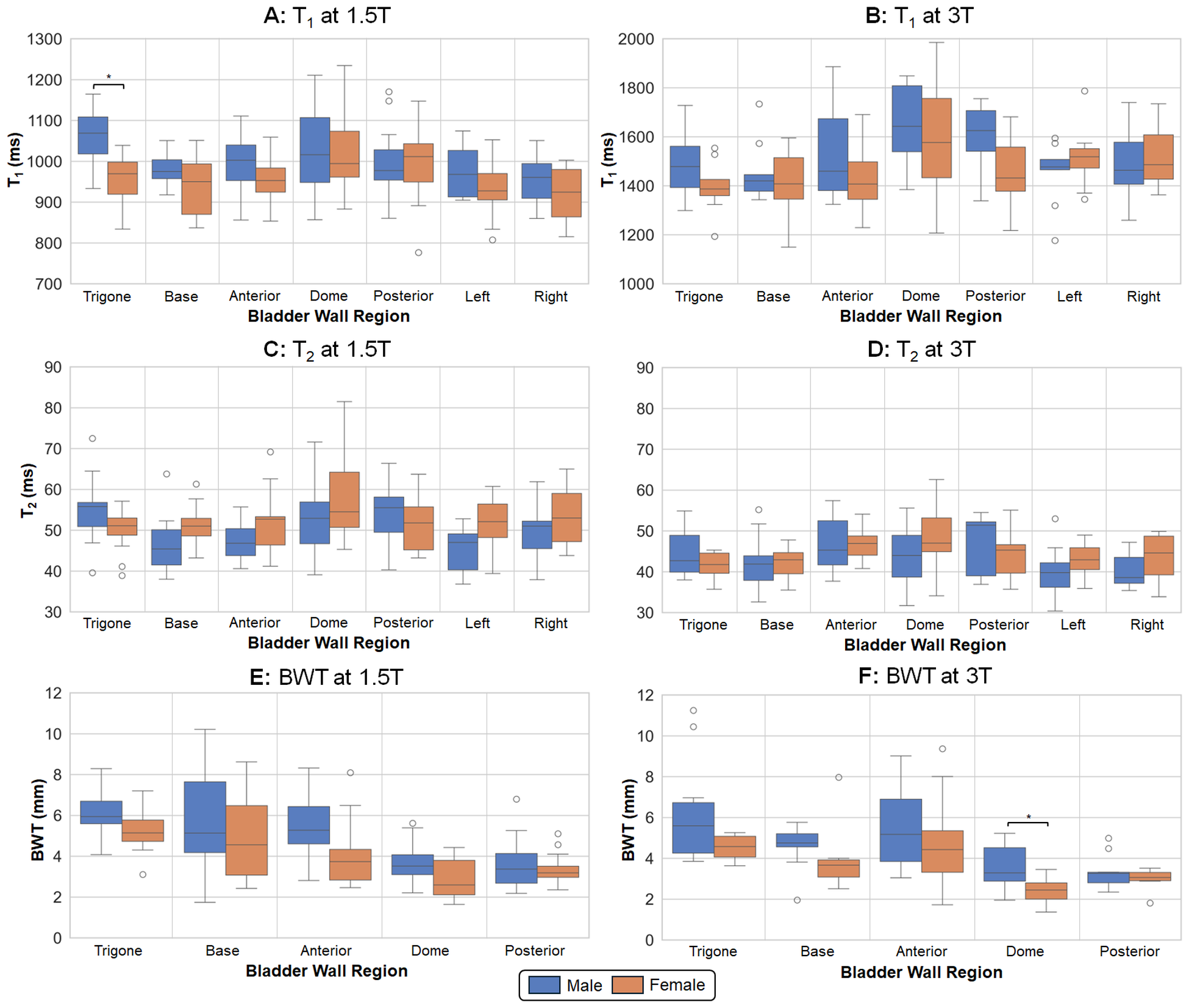


**Supporting Figure S11:** ***Comparison of MRF relaxation times and BWT in male and female subjects.*** Regional measurements of **(A, B)** T_1_, **(C, D)** T_2_, and **(E, F)** BWT acquired with MRF at 1.5T and 3T are presented for male and female subjects. For each bladder region, significant sex differences are indicated by an asterisk (* *p* < 0.05, ** *p* < 0.01, *** *p* < 0.001).

# 11. Associations between Regional MRF Measurements and Age


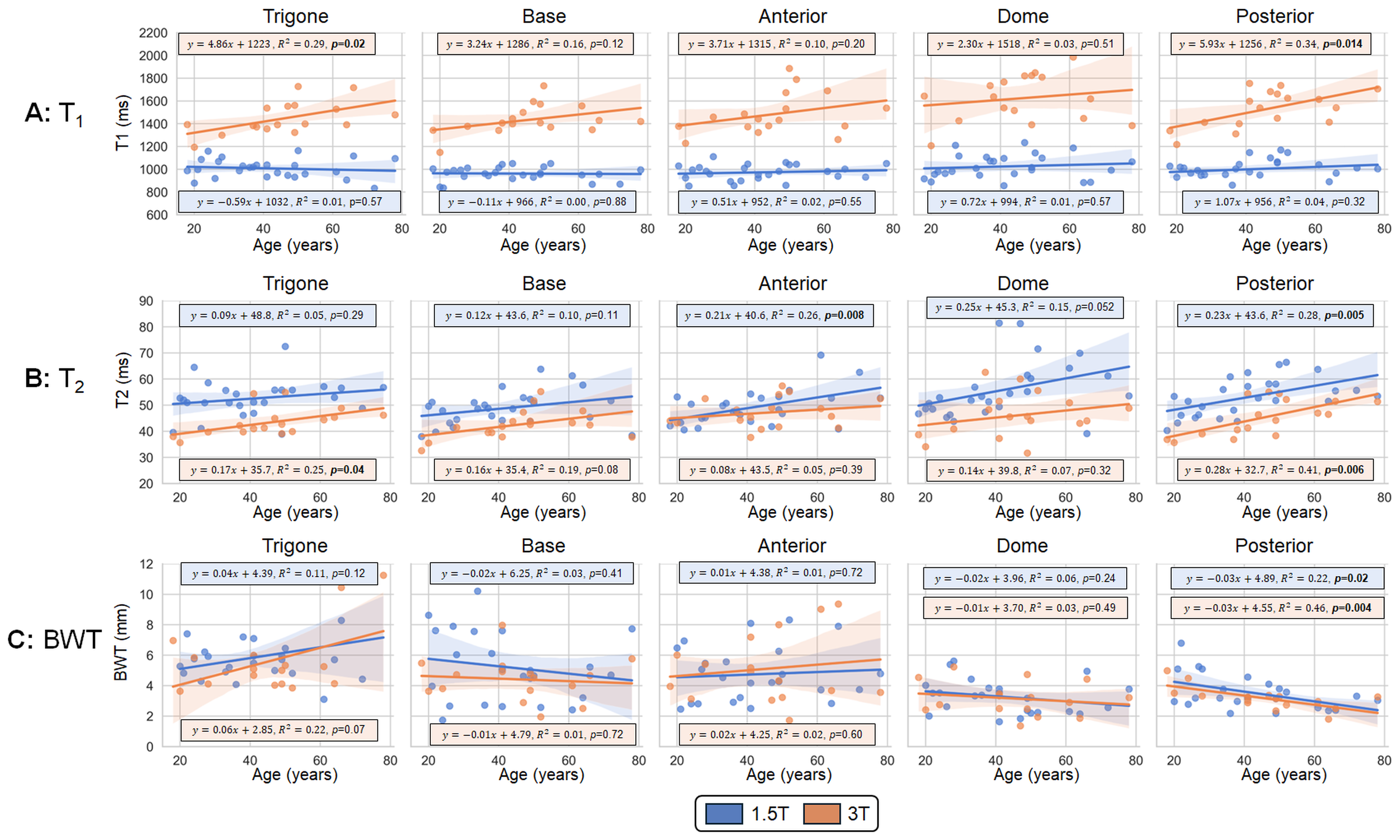


**Supporting Figure S12: *Association between bladder MRF relaxation times and BWT with age.*** Regional **(A)** T_1_, **(B)** T_2_, and **(C)** BWT measurements using MRF at 1.5T and 3T are displayed as a function of age. The best-fit regression line is reported for each case, along with the coefficient of determination (R^2^ value) and *p*-value. Shaded areas indicate the 95% confidence interval for the regression.

# 12. Potential Explanation of Bright Rim Artifact on 3T MRF T_1_ Maps

A bright rim of elevated values was consistently observed along the outer bladder wall contour on the 3T MRF T_1_ maps. This artifact was not apparent in the T_2_ maps and did not occur at 1.5T. An example is provided in **Supporting Figure S13**, and the artifact is also visible in **Figures 2-3** and **Supporting Figures** **S3-S6**. We believe the most likely source is signal contamination from fat at fat-water interfaces. This interpretation is supported by two observations. First, the artifact is present at 3T but not at 1.5T, consistent with the larger fat frequency offset at higher field. Second, it occurs where the bladder wall is adjacent to perivesical fat; it is not seen at the base of the bladder adjacent to the prostate in men, where no perivesical fat is present. The MRF sequence uses a 3.5 ms spiral readout without fat suppression or off-resonance correction. Under these conditions, off-resonant fat signal can blur into adjacent bladder wall voxels and corrupt the measured fingerprint. A potentially counterintuitive aspect is that fat has a shorter T_1_ than bladder wall, so one might expect a reduced T_1_ due to partial volume averaging. However, in MRF, when a mixed fat/water signal is matched to a water-only dictionary, the direction of the resulting error is not straightforward to predict analytically.

To test whether this mechanism could plausibly lead to the rim artifact, we performed a simulation in which on-resonant bladder wall and off-resonant fat fingerprints were combined at varying fat fractions and then matched to the dictionary used in the study, which neglected off-resonance effects. At 3T, the simulation used an on-resonant bladder wall fingerprint (T_1_ = 1400 ms, T_2_ = 40 ms) and an off-resonant fat fingerprint (-440 Hz, T_1_ = 350 ms, T_2_ = 80 ms). The combined fingerprint was evaluated over fat fractions from 0% to 100%. As shown in **Supporting Figure S14**, the apparent T_1_ was markedly overestimated at low-to-moderate fat fractions, reaching the upper bound of the dictionary (4000 ms) at approximately 20–25% fat fraction, whereas the apparent T_2_ changed less over the same range. This behavior is consistent with the observed artifact, having a bright T_1_ rim at the bladder-fat interface but less conspicuous abnormalities on the T_2_ map. At 1.5T, the simulation used bladder wall values of T_1_ = 1000 ms and T_2_ = 50 ms, and an off-resonant fat fingerprint at -220 Hz (T_1_ = 260 ms, T_2_ = 85 ms). In this case, the apparent T_1_ decreased monotonically with increasing fat fraction, consistent with the absence of a rim artifact.

**Supporting Figure S13: Bright rim artifact on a 3T MRF T_1_ map.** Representative MRF T_1_ and T_2_ maps from the same subject are shown at **(A)** 1.5T and **(B)** 3T. On the 3T T_1_ map, a bright rim of elevated values is visible at the interface between the bladder wall and perivesical fat (arrows). This artifact is not apparent on the 3T T_2_ map or the 1.5T T_1_ and T_2_ maps.

**Supporting Figure S14:** ***Effect of partial volume averaging from fat on MRF bladder wall relaxation time measurements.*** Simulation results show **(A, B)** the estimated apparent T_1_ and T_2_ at 1.5T and **(C, D)** at 3T. Dotted lines indicate the simulated reference T_1_ and T_2_ values for pure bladder wall (blue) and pure fat (green).
